# Seven New Alkaloids Isolated from Marine Flavobacterium *Tenacibaculum discolor* sv11

**DOI:** 10.3390/md20100620

**Published:** 2022-09-30

**Authors:** Lei Wang, Michael Marner, Ute Mettal, Yang Liu, Till F. Schäberle

**Affiliations:** 1 Institute for Insect Biotechnology, Justus-Liebig-University Giessen, 35392 Giessen, Germany; 2 Fraunhofer Institute for Molecular Biology and Applied Ecology (IME), Branch for Bioresources, 35392 Giessen, Germany; 3 German Center for Infection Research (DZIF), Partner Site Giessen-Marburg-Langen, 35392 Giessen, Germany

**Keywords:** Bacteroidetes, *Tenacibaculum*, nitrogen-containing heterocycles, imidazolium-containing alkaloids, pyridinium-containing alkaloid, antimicrobial activity

## Abstract

Marine flavobacterium *Tenacibaculum discolor* sv11 has been proven to be a promising producer of bioactive nitrogen-containing heterocycles. A chemical investigation of *T.* *discolor* sv11 revealed seven new heterocycles, including the six new imidazolium-containing alkaloids discolins C-H (**1**–**6**) and one pyridinium-containing alkaloid dispyridine A (**7**). The molecular structure of each compound was elucidated by analysis of NMR and HR-ESI-MS data. Furthermore, enzymatic decarboxylation of tryptophan and tyrosine to tryptamine and tyramine catalyzed by the decarboxylase DisA was investigated using in vivo and in vitro experiments. The antimicrobial activity of the isolated compounds (**1**–**7**) was evaluated. Discolin C and E (**1** and **3**) exhibited moderate activity against Gram-positive *Bacillus subtilis* DSM10, *Mycobacterium smegmatis* ATCC607, *Listeria monocytogenes* DSM20600 and *Staphylococcus aureus* ATCC25923, with MIC values ranging from 4 μg/mL to 32 μg/mL.

## 1. Introduction

Structurally diverse nitrogen-containing heterocycles, such as pyrroles, imidazoles, oxazoles, pyridines, and quinolones, are widely distributed in marine organisms and microorganisms. These naturally occurring secondary metabolites often exhibit significant pharmacological activities, including antibacterial, antifungal, antiparasitic, and anticancer activities [1,2,3,4,5,6]. Furthermore, these compounds are often amenable to further structural modifications [7,8,9,10]. Currently, marine-derived imidazole alkaloids are reported mainly to be isolated from sponges, while reports of marine bacteria as bioresource are relatively rare [2,6,11,12,13,14].

As a member of the family *Flavobacteriaceae* within the phylum *Bacteroidetes*, isolates of the genus *Tenacibaculum* have been mainly obtained from marine environments, such as sea water, tidal flat, and aquaculture systems, as well as marine organisms like bryozoan, sea anemone, oyster, sponge and green algae [15,16,17,18,19,20,21,22]. Bacteria of this genus are the etiological agent of an ulcerative disease known as tenacibaculosis, which affects a large number of marine fish species in the world [23]. Up to now, the natural products isolated from *Tenacibaculum* strains comprise only siderophores that showed beside their chelating activity also cytotoxicity [24,25,26], and phenethylamine-containing heterocycles. The latter include two imidazole alkaloids identified in our previous search for antimicrobial metabolites from marine flavobacteria. It was shown that they could be synthesized by decarboxylation of phenylalanine, catalyzed by the enzyme DisA [27]. Likewise, the tryptamine and phenethylamine moieties of imidazole alkaloids isolated from a marine sponge-associated *Bacillus* strain were proposed to be formed by an aromatic amino acid decarboxylase-dependent reaction [28]. In order to further expand the array of available nitrogen-containing heterocycles, the metabolome of *T. discolor* sv11 was further investigated. Herein, we present the isolation, structure elucidation and biological activity of new alkaloids from the bacterium, and link the enzymatic activity of DisA to their biosynthesis using both, in vivo and in vitro assays.

## 2. Results

**Figure 1:**
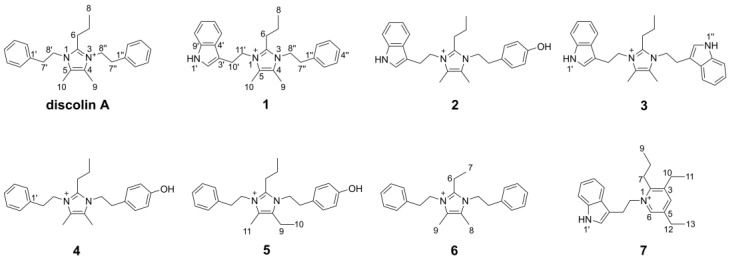
Discolin A and new compounds isolated from *T. discolor* sv11.

In our continuous search for new bioactive molecules, the six new imidazolium-containing alkaloids discolins C–H (**1**–**6**) and one pyridinium-containing alkaloid dispyridine A (**7**) were isolated from the marine-derived bacterium *T. discolor* sv11 (Figure 1). The antimicrobial activity of these new compounds was investigated, among which, compounds **1** and **3** exhibited moderate activity against Gram-positive *Bacillus subtilis* DSM10, *Mycobacterium smegmatis* ATCC607, *Listeria monocytogenes* DSM20600 and *Staphylococcus aureus* ATCC25923. In vivo and in vitro experiments indicated that phenethylamine, tryptamine and tyramine residues of the new alkaloids are derived from an enzymatic decarboxylation.

**Figure 2:**
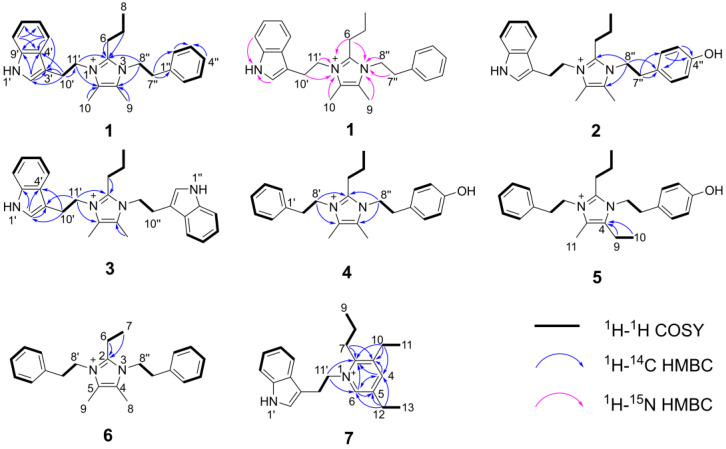
Key HMBC and ^1^H-^1^H COSY correlations of compounds **1**–**7**.

Compound **1** was obtained as a yellowish oil. The HR-ESI-MS spectrum of **1** showed a molecular formula of C_26_H_32_N_3_^+^ based on the prominent peak [M]^+^ at *m/z* 386.2606 (calculated 386.2591, Figure S1). The analysis of ^1^H NMR and HSQC spectra of **1** revealed three methyl groups at *δ*_H_ 0.80 (H-8), *δ*_H_ 2.15 (H-9) and *δ*_H_ 2.20 (H-10), six methylene groups at *δ*_H_ 1.34 (H-7), *δ*_H_ 2.41 (H-6), *δ*_H_ 2.80 (H-7″), *δ*_H_ 3.08 (H-10′), *δ*_H_ 4.22 (H-8″) and *δ*_H_ 4.30 (H-11′), as well as ten aromatic protons that resonated from *δ*_H_ 7.00 to *δ*_H_ 7.39 (Table 1). These NMR data exhibited a high similarity with the previously reported compound discolin A that was also isolated from *T*. *discolor* sv11 [27]. Therefore, a core structure of the 4,5-dimethyl-2-propyl imidazolium skeleton of **1** was elucidated based on the COSY spin system from H-6 to H-7 to H-8, as well as on HMBC correlations from both H-6 and H-7 to C-2, and from both, H-9 and H-10 to C-4 and C-5. The same phenylethyl moiety as present in discolin A was deducted from compound **1** based on the COSY spin system between H-7″ and H-8″, as well as between five benzene ring protons 7.16 (2H, H-2″ and H-6″), 7.28 (H-4″) and *δ*_H_ 7.32 (2H, H-3″ and H-5″), together with the core HMBC correlations from H-7″ to C-1″ and C-2″, and from H-8″ to C-1″ (Figure 2). The significant difference between compound **1** and discolin A are the HMBC correlations from a singlet aromatic proton resonating at *δ*_H_ 7.17 (H-2′) to C-3′, C-4′ and C-9′, from H-5′ to C-3′, C-4′, C-7′ and C-9′ (Figure 2), as well as the COSY spin system from H-5′ to H-6′, to H-7′ to H-8′. These results suggested an indole moiety instead of a phenyl residue in compound **1**. Together with the remaining COSY spin system between the two methylene groups H-10′ and H-11′ and the HMBC correlations from H-10′ to C-2′, C-3′, C-4′ and C-11′, a 3-ethylindole moiety (C_10_H_10_N) was elucidated from compound **1**, which is further supported by the MS/MS fragment [C_10_H_10_N]^+^ detected at *m/z* 144.0813 (calculated 144.0813, Figure S1). With the HMBC correlations from H-8″ to C-2 and C-4, and from H-11′ to C-2 and C-5, the above mentioned phenylethyl moiety and 3-ethylindole were supposed to be located at position 3 and 1 of the imidazolium skeleton (Figure 2). This assumption was proven by ^1^H-^15^N HMBC correlations from H-6, H-9, H-7″ and H-8″ to N-3 and from H-6, H-10, H-10′ and H-11′ to N-1 (Figure 2). The N-atom at position 1 was considered to be positively charged based on the detected chemical shift at *δ*_N_ 178.5, while N-3 was at *δ*_N_ 177.3 (Figures S7 and S8) [29,30,31]. An additional NMR measurement with added trifluoracetic acid (TFA) in DMSO-*d_6_* (ratio 1:3) was carried out to further prove this conclusion (Figures S9–S13). The methylene groups of H-8″ and H-7″ shifted to up-field with a deviation Δ*δ*_H-8″_ value of 0.13 and Δ*δ*_H-7″_ value of 0.10 ppm, while the deviation Δ*δ*_H-11′_ and Δ*δ*_H-10′_ values were 0.09 and 0.05 ppm, respectively. The addition of TFA lead to the protonation of the tertiary N-atom at position 3, which gives a higher influence on the chemical shift [32]. The detected different chemical shift deviations Δ*δ*_N-3_ (155.1) and Δ*δ*_N-1_ (154.1) further support this result (Figures S7, S8 and S13). Thus, the structure of compound **1** was elucidated as shown in Figure 1 and named discolin C.

Compound **2** was obtained as a yellowish oil. The molecular formula of **2** was determined to be C_26_H_32_ON_3_^+^ (*m/z* = 402.2543, [M]^+^, calcd. 402.2540, Figure S14) based on the HR-ESI-MS spectrum. Comprehensive comparison of NMR data of compounds **1** and **2** revealed the high similarity except for the chemical shift of C-4″, which was shifted from 126.96 to 156.48, and one missing aromatic proton. Together with the detected 16 Da increase in the HR-ESI-MS spectrum of compound **2**, this suggested the presence of a 4-hydroxyphenylethyl moiety located at position 3 instead of a phenylethyl moiety (Table 1). This assumption was confirmed by the upfield chemical shifts of the benzene ring protons at *δ*_H_ 6.70 (2H, H-3″ and H-5″) and *δ*_H_ 6.91 (2H, H-2″ and H-6″), which showed similar behavior as the reported 4-hydroxyphenylethyl-containing compound *N*-Acetyltyramine [33]. This effect is explained by the fact that the hydroxyl group is an electron donor, which shields the protons of the benzene nucleus more strongly and leads to an upfield shift of the corresponding signals. Second, the hydroxyl group that appeared at C-4″ in compound **2** changes the spin system of this radical, and therefore influenced the shape of the proton multiplets of the benzene nucleus. Furthermore, the COSY spin system between H-2″ and H-3″, as well as the HMBC correlations from H-7″ to C-1″, C-2″, from H-3″ to C-1″, C-4″, and from H-8″ to C-2, C-4 and C-1″ proved the 4-hydroxyphenylethyl group in compound **2**. The detected MS/MS fragment at *m/z* 282.1963 ([C_18_H_23_N_3_ + H]^+^, calcd. 282.1965, Figure S14) of compound **2**, which lost the 4-hydroxyphenylethyl group (-C_8_H_9_O), strongly indicated the above mentioned assumption. Thus, the structure of compound **2** was elucidated as shown in Figure 1 and named discolin D.

Compound **3** was also obtained as a yellowish oil. The molecular formula of **3** was determined to be C_28_H_33_N_4_^+^ (*m/z* = 425.2702, [M]^+^, calculated 425.2700, Figure S20) based on the HR-ESI-MS spectrum. The core scaffold of compound **3** shared the 4,5-dimethyl-2-propyl imidazolium skeleton with compound **1** as deduced from a comparison of both, 1D and 2D NMR data (Table 1), which also indicated compound **3** to be a symmetric structure. Integration of the proton signals in the ^1^H NMR spectrum together with the COSY spin system of the aromatic protons and the HMBC correlations from H-2′ to C-3′, C-4′ and C-9′, from H-10′ to C-2′, C-4′ and C-11′, as well as from H-11′ to C-3′, C-10′, C-2, and C-5 proved that two identical 3-ethylindole moieties were connected to the central imidazolium ring as shown in Figure 2. Hence, compound **3** is a member of the discolin family and was named discolin E.

Compounds **4** and **5** were each obtained as a yellowish oil. The molecula formulae of compounds **4** and **5** were identified as C_24_H_31_ON_2_^+^ and C_25_H_33_ON_2_^+^, respectively, based on the HR-ESI-MS signals [M]^+^ at *m/z* = 363.2442 (calculated 363.2431, compound **4**, Figure S26) and *m/z* = 377.2593 (calculated 377.2587, compound **5**, Figure S32), respectively. One phenylethyl moiety, one 4-hydroxyphenylethyl moiety and the 4,5-dimethyl-2-propyl imidazolium skeleton were disclosed as constituents of compound **4** by comparing the 1D and 2D NMR data with those of compounds **1** and **2** (Table 1 and Table 2). The phenylethyl moiety and the 4-hydroxyphenylethyl moiety were assigned to be located at positions 1 and 3 of the imidazolium skeleton of compound **4**, based on the HMBC correlations from H-8′ to C-2 and C-5 and from H-8″ to C-2 and C-4 (Figure 2). In compound **5**, identical phenylethyl and 4-hydroxyphenylethyl moieties were assigned to be located at the same positions as in compound **4**. Comparing the 1D and 2D NMR data of compounds **4** and **5**, the only difference is one ethyl group present in compound **5**, while compound **4** carries a methyl group (Table 2). The presence of an ethyl group in compound **5** is corroborated by the COSY correlation between H-9 and H-10, and the HMBC correlations from both, H-9 and H-10 to C-4. In contrast, in compound **4**, the methyl group is directly connected to the unsaturated carbon C-4 (Figure 2), thus verifying the assumed structural relationship between compounds **4** and **5**. The 14 Da molecular weight difference between both compounds further supports their structural relationship. Thus, the structures of compounds **4** and **5** were elucidated as shown in Figure 1, and the names discolin F and discolin G were proposed, respectively.

Compound **6** was also obtained as a yellowish oil. The molecular formula of compound **6** was established as C_23_H_29_N_2_^+^ based on the prominent [M]^+^ peak in HR-ESI-MS spectrum at *m/z* = 333.2329 (calculated 333.2325, Figure S37). Two identical phenylethyl moieties were deduced from the NMR spectra of compound **6** and connected at positions 1 and 3 of the core ring based on the HMBC correlations from H-8′ to C-2 and C-5, as well as from H-8″ to C-2 and C-4. The remaining signals of **6** were assigned to the 4,5-dimethyl-2-ethyl imidazolium scaffold, which showed an ethyl group rather than a propyl group at position 2. This difference was clarified by the COSY correlation between H-6 and H-7, as well as the HMBC correlations from both, H-6 and H-7 to C-2 (Figure 2). Therefore, compound **6** proved to be a representative of the discolin family and was named discolin H.

Compound **7** was isolated as a colorless powder. Its molecular formula was established as C_22_H_29_N_2_^+^ based on the prominent ion peak [M]^+^ observed at *m/z* 321.2322 (calcd. 321.2325, Figure S43). Comprehensive analysis of 1D and 2D NMR data of compound **7** revealed one 3-ethylindole moiety as found in compounds **1**–**3**, as well as two ethyl groups and one propyl group (Table 3). The remaining two aromatic protons at *δ*_H_ 8.20 (H-4) and *δ*_H_ 8.40 (H-6) in the ^1^H NMR spectrum and five aromatic carbons at *δ*_C_ 153.07 (C-2), *δ*_C_ 142.58 (C-3), *δ*_C_ 144.54 (C-4), *δ*_C_ 140.61 (C-5) and *δ*_C_ 142.62 (C-6) in the ^13^C NMR spectrum were attributed to a pyridinium ring as apparent from comparison with the data of dispyridine, a pyridinium-containing alkaloid isolated previously [27]. The location of the 3-ethylindole moiety was determined from the HMBC correlations from H-11′ to C-2 and C-6, which also confirmed the location of the aromatic proton H-6 resonating at *δ*_H_ 8.40. The second aromatic proton resonating at *δ*_H_ 8.20 was attributed to position 4, based on the HMBC correlations from H-4 to C-2, C-3 and C-6. The propyl group and the two ethyl groups attached to the pyridinium ring were located at C-2, C-3 and C-5, as inferred from HMBC correlations from H-7 to C-2 and C-3, from H-10 to C-2, C-3 and C-4, and from H-12 to C-4, C-5 and C-6 (Figure 2). Therefore, compound **7** was found to be a new member of the dispyridine family and named dispyridine A.

**Figure 3:**
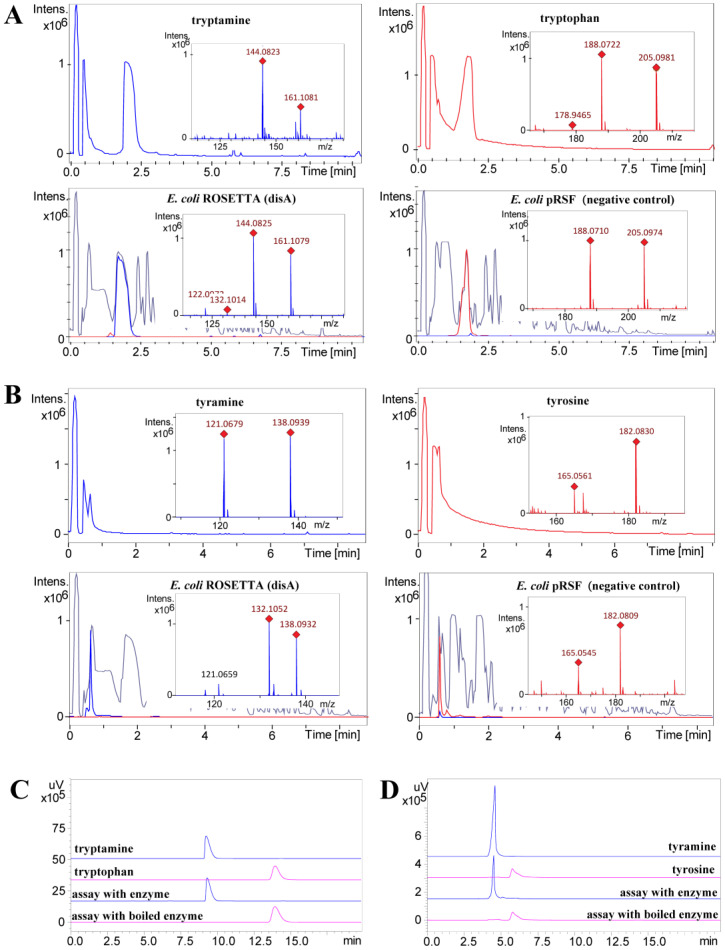
In vivo and in vitro decarboxylation of tryptophan and tyrosine catalyzed by DisA. (**A**) In vivo decarboxylation of tryptophan to tryptamine. Extracted ion chromatograms (EICs) of tryptophan (C_11_H_13_N_2_O_2_ 205.0977 [M + H]^+^, in red) and tryptamine (C_10_H_13_N_2_ 161.1079 [M + H]^+^, in blue). (**B**) In vivo decarboxylation of tyrosine to tyramine. Extracted ion chromatograms (EIC) of tyrosine (C_9_H_12_NO_3_ 182.0817 [M + H]^+^, in red) and tyramine (C_8_H_12_NO 138.0919 [M + H]^+^, in blue). In A and B, the base peak chromatogram of the extracts is given in grey. (**C**) Comparative HPLC analysis of the in vitro decarboxylation of tryptophan. (**D**) Comparative HPLC analysis of the in vitro decarboxylation of tyrosine. In C and D, the UV absorbance at 254 nm is shown.

Based on previous research, it was known that phenylalanine can be converted to phenethylamine by the catalytic action of the decarboxylase DisA. This molecule could serve as building block to yield different derivatives [27]. Hence, the newly isolated imidazolium-containing alkaloids (**1**–**6**) were also supposed to be produced via the same biosynthetic route, i.e., first an enzymatic decarboxylation of the aromatic-*L*-amino acid tryptophan or tyrosine yielding tryptamine or tyramine, respectively, followed by a non-enzymatic condensation to form the central imidazolium ring. To confirm this hypothesis, the candidate enzyme DisA from *T. discolor* sv11 was analyzed in vivo in a heterologous system. Therefore, the previously constructed transgenic host strain *E. coli* ROSETTA (disA) carrying the respective *disA* gene and *E. coli* ROSETTA (pRSF) (negative empty vector control) were cultivated in LB medium, whereby 2 mM tryptophan and tyrosine were added as substrates, respectively. After 24 h incubation, tryptamine and tyramine were only detected in the extract of *E. coli* ROSETTA (disA), while only the substrates, i.e., tryptophan and tyrosine were detected in the negative control (Figure 3). To further validate these results, a His-tagged version of DisA was purified using affinity chromatography and assayed in vitro. This confirmed that tryptamine and tyramine can be obtained from tryptophan and tyrosine by a DisA-dependent catalytic conversion (Figure 3).

All isolated compounds **1**–**7** were investigated for their bioactivity against bacteria (*B. subtilis* DSM10, *M. smegmatis* ATCC607, *L. monocytogenes* DSM20600, *S. aureus* ATCC25923, and *E. coli* ATCC25922) and fungi (*Candida albicans* FH2173). As shown in Table 4, discolin C (**1**) showed activity against *M. smegmatis* ATCC607 and *B. subtilis* DSM10 with MIC values ranging from 4 μg/mL to 8 μg/mL and moderate to weak activity against *S. aureus* ATCC25923 and *L. monocytogenes* DSM20600 with MIC values ranging from 16 μg/mL to 32 μg/mL. Discolin E (**3**) exhibited activity against four tested Gram-positive bacteria with MIC values ranging from 4 μg/mL to 8 μg/mL and moderate activity against *C. albicans* FH2173 with an MIC value of 16 μg/mL. The other compounds (**2**, **4**–**7**) were inactive against all the tested microorganisms in the range tested.

## 3. Conclusions and Discussion

In conclusion, seven new alkaloids were obtained from the crude extract of *T. discolor* sv11 after fermentation in LB medium. In vivo and in vitro experiments proved the decarboxylase DisA to catalyze the decarboxylation of the aromatic-*L*-amino acids phenylalanine, tryptophan and tyrosine to phenethylamine, tryptamine and tyramine, respectively. These molecules serve as substrates for the formation of the central imidazolium ring of discolins A–H through a non-enzymatic condensation. Hence, by combining an enzyme-catalyzed and a non-enzymatic reaction, the bacterium generates a mix of structurally related molecules. Besides the understanding of the biosynthetic mechanisms of the discolins, some insights into the structure–activity relationship of the antibacterial discolins A-H were also obtained [27]. Discolin A and discolin H feature the same molecular skeleton, except for the length of the carbon chain linked to C-2 of the central imidazolium ring. Both molecules showed the similar moderate bioactivity, which suggests that the substructure at position 2 can be altered without affecting the activity. The structural differences and changes in the bioactivity of discolins C-F and discolin A indicated that the substructures at position 1 and 3 of the central ring instead play an important role concerning antibacterial activity. Earlier evidence indicated that the activity of imidazolium salts is highly dependent upon the substituents on the nitrogen atoms of the imidazolium cation [34], which is in agreement with our observation. In summary, our finding, together with previous reports, clearly indicates that the genus *Tenacibaculum* exhibits a high potential to produce nitrogen-containing heterocycles with a unique structure and various biological activities. This includes positively charged imidazolium-containing natural products.

## 4. Materials and Methods

### 4.1. General Experimental Procedures

The 1D and 2D NMR spectra were recorded in DMSO-*d*_6_ using a Bruker Avance Neo 700 MHz spectrometer equipped with a 5 mm CryoProbe Prodigy TCI (^1^H,^15^N,^13^C Z-GRD) (Bruker, Ettlingen, Germany). The LC-HRMS data for new compounds were recorded on a micrOTOF-QII mass spectrometer (Bruker, Billerica, MA, USA) equipped with an ESI-source coupled to an Agilent Infinity 1290 UHPLC system using an ACQUITY UPLC BEH C18 Column, 130 Å, 1.7 µm, 2.1 mm×100 mm (Waters, Eschborn, Germany) with an ACQUITY UPLC BEH C18 VanGuard Pre-column, 130 Å, 1.7 µm, 2.1 mm × 5 mm (Waters, Eschborn, Germany). HPLC was performed using a Shimadzu HPLC system (Shimadzu Deutschland GmbH, Duisburg, Germany) for analysis (EC 250/4.6 Nucleodur C18 Gravity-SB, 5 μm; Macherey-Nagel, Düren, Germany), and for semi-preparative purification (VP 250/10 Nucleodur C18 Gravity-SB, 5 μm; Macherey-Nagel, Düren, Germany). MPLC was performed on the Interchim Puriflash 4125 chromatography system (Interchim, Montluçon, France).

### 4.2. Extraction and Isolation

A fermentation (36 L) of *T. discolor* S11 was performed in 5 L flasks that contained 1.5 L of LB medium and were incubated at 30 °C and 140 rpm for 8 days, followed by an extraction using EtOAc (volume ratio 1:1) for three times, affording 11.9 g crude extract. Thirteen fractions (Fr. 1–13) were collected from reversed phase flash chromatography (Interchim Puriflash 4125 chromatography system with Puriflash C18-AQ30 μm F0120 column) with an elution gradient starting from 10% MeOH/H_2_O to 100% MeOH over 1.5 h. Fr. 11 (973.8 mg) was further subjected to size exclusion chromatography on a Sephadex LH-20 column and eluted with 100% MeOH to give 10 subfractions (Frr. 11.1–11.10). Frr. 11.4 (229.4 mg) was further subjected to reversed phase flash chromatography (Interchim Puriflash 4125 chromatography system with Puriflash C18-HP30 μm F0025 Flash column) using an elution gradient from 10% MeOH/H_2_O to 100% MeOH over 4 h to give 10 subfractions (Frrr. 11.4.1–11.4.10). Frrr. 11.4.6 was further purified by semi-preparative HPLC (0–1 min, 22% MeCN; 1–46 min, gradient increased from 22% to 37% MeCN) to yield compounds **1** (2.0 mg, *t*_R_ = 43 min) and **3** (0.6 mg, *t*_R_ = 45 min). Frrr. 11.4.5 was fractionated by semi-preparative HPLC (0–38.5 min, gradient increased from 10% to 46% MeOH) to give 6 subfractions (Frrrr. 11.4.5.1–11.4.5.6). Frrrr. 11.4.5.3 was again purified by semi-preparative HPLC (0–57 min, isocratic gradient with 29% MeOH) to yield compounds **2** (1.2 mg, *t*_R_ = 48.5 min), **4** (2.1 mg, *t*_R_ = 39 min) and **7** (1.5 mg, *t*_R_ = 44.4 min). Purification of Frrrr. 11.4.5.1 by semi-preparative HPLC (0–5 min, 5% MeCN; 5–50 min, gradient increased from 5% to 35% MeCN) yielded compound **5** (0.3 mg, *t*_R_ = 49.3 min). Compound **6** (0.3 mg, *t*_R_ = 51.2 min) was obtained from Frrrr. 11.4.5.6 by semi-preparative HPLC (0–5 min, 5% MeCN; 5–56 min, gradient increased from 5% to 39% MeCN).

Discolin C (**1**): yellowish oil; the ^1^H NMR (DMSO-*d*_6_, 700 MHz) and ^13^C NMR (DMSO-*d*_6_, 175 MHz) data are given in Table 1; HR-ESI-MS *m/z* 386.2606 [M]^+^ (calculated for C_26_H_32_N_3_^+^, 386.2591, Figure S1).

Discolin D (**2**): yellowish oil; the ^1^H NMR (DMSO-*d*_6_, 700 MHz) and ^13^C NMR (DMSO-*d*_6_, 175 MHz) data are given in Table 1; HR-ESI-MS *m/z* 402.2543 [M]^+^ (calculated for C_26_H_32_ON_3_^+^, 402.2540, Figure S14).

Discolin E (**3**): yellowish oil; the ^1^H NMR (DMSO-*d*_6_, 700 MHz) and ^13^C NMR (DMSO-*d*_6_, 175 MHz) data are given in Table 1; HR-ESI-MS *m/z* 425.2702 [M]^+^ (calculated for C_28_H_33_N_4_^+^, 425.2700, Figure S20).

Discolin F (**4**): yellowish oil; the ^1^H NMR (DMSO-*d*_6_, 700 MHz) and ^13^C NMR (DMSO-*d*_6_, 175 MHz) data are given in Table 2; HR-ESI-MS *m/z* 363.2442 [M]^+^ (calculated for C_24_H_31_ON_2_^+^, 363.2431, Figure S26).

Discolin G (**5**): yellowish oil; the ^1^H NMR (DMSO-*d*_6_, 700 MHz) and ^13^C NMR (DMSO-*d*_6_, 175 MHz) data are given in Table 2; HR-ESI-MS *m/z* 377.2593 [M]^+^ (calculated for C_25_H_33_ON_2_^+^, 377.2587, Figure S32).

Discolin H (**6**): yellowish oil; the ^1^H NMR (DMSO-*d*_6_, 700 MHz) and ^13^C NMR (DMSO-*d*_6_, 175 MHz) data are given in Table 2; HR-ESI-MS *m/z* 333.2329 [M]^+^ (calculated for C_23_H_29_N_2_^+^, 333.2325, Figure S37).

Dispyridine A (**7**): colorless powder; the ^1^H NMR (DMSO-*d*_6_, 700 MHz) and ^13^C NMR (DMSO-*d*_6_, 175 MHz) data are given in Table 3; HR-ESI-MS *m/z* 321.2322 [M]^+^ (calculated for C_22_H_29_N_2_^+^, 321.2325, Figure S43).

### 4.3. Enzymatic Activity of Dis A

To investigate the enzymatic activity of Dis A in vivo, *E. coli* ROSETTA (disA) was cultured in 30 mL kanamycin-containing (50 μg mL^−1^) LB medium at 30 °C overnight as pre-culture. A volume of 100 μL of this pre-culture was used to inoculate at 37 °C in two 300 mL Erlenmeyer flasks with 100 mL kanamycin-containing (50 μg mL^−1^) LB medium; 0.1 mM IPTG was added into the medium when the cultures reached an OD_600_ of 0.5 and were cultured at 30 °C for 3 h. Then, 2 mM tryptophan or tyrosine were added to the medium and cultured at 30 °C overnight. Next, 2 mL medium was harvested, dried *in vacuo*, re-dissolved in 200 μL DMSO and analyzed by UPLC-HRMS. The *E. coli* ROSETTA strain harboring the empty vector pRSF without the target *disA* gene was cultivated under the same conditions and analyzed by UPLC-HRMS as the negative control.

An in vitro enzymatic characterization was carried out after the purification of the His-tagged DisA. An inoculum of 15 mL of same pre-culture prepared for in vivo assay was used to inoculate 1.5 L kanamycin-containing (50 μg mL^−1^) LB medium; 0.1 mM IPTG was added to the medium when the cultures reached an OD_600_ of 0.5 and were cultured overnight. Cells were collected by centrifugation at 4 °C with 10,000 rpm and resuspended in lysis buffer (50 mM NaH_2_PO_4_, 300 mM NaCl and 10 mM imidazole; pH 8.0). The resulting suspensions were sonicated and centrifuged at 4 °C at maximum speed for 30 min. The supernatant was loaded onto a pre-equilibrated 750 μL Qiagen^®^ Ni-NTA column. After washing with a 3 mL lysis buffer and 3 mL wash buffer (20 mM imidazole lysis buffer), the His-tagged protein DisA was eluted from the column using an elution buffer (250 mM imidazole lysis buffer) (Figure S49). The protein was resuspended into an imidazole-free buffer (50 mM NaH_2_PO_4_, 300 mM NaCl; pH 8.0) and concentrated using the Amicon^®^ Ultra-15 centrifugation membrane column.

Enzymatic reactions were performed in 50 mM lysis buffer without imidazole (50 mM NaH_2_PO_4_, 300 mM NaCl, pH 8.0), containing 100 μM tryptophan (or 20 μM tyrosine) and 5 μM DisA in a total volume of 0.5 mL. After incubation at 30 °C overnight, the same volume of MeOH was added to quench the reactions. The reaction mixture was then centrifuged and the supernatant was dried and re-dissolved in 50 μL 50% MeOH and analyzed by analytical HPLC (0–16 min, 5% MeCN; 16–26 min, gradient increased from 5% to 100% MeCN).

### 4.4. Bioactivity Tests

Determination of the minimum inhibitory concentration (MIC) of purified compounds **1**–**7** was carried out by micro broth dilution assays in 96 well plates as described previously [27]. All compounds were dissolved in dimethyl sulfoxide (DMSO, Carl Roth GmbH + Co., Karlsruhe, Germany) with a concentration of 3.2 mg/mL and tested in triplicate. Dilution series (64−0.03 μg/mL) of rifampicin, tetracycline, and gentamicin (all Sigma-Aldrich, St. Louis, MS, USA) were prepared as positive controls for *B. subtilis* DSM10, *L. monocytogenes* DSM20600, *S. aureus* ATCC25923, and *E. coli* ATCC25922. Same dilution series of rifampicin, tetracycline, and isoniazid for *M. smegmatis* ATCC607. For fungi (*C. albicans* FH2173), tebuconazole (Cayman Chemical Company, Ann Arbor, MI, USA.), amphotericin B (Sigma-Aldrich, St. Louis, MS, USA) and nystatin (Sigma-Aldrich, St. Louis, MS, USA) were used as the positive control with same dilution series.

## Figures and Tables

**Table 1:** ^1^H (700 MHz) and ^13^C (175 MHz) NMR data of compounds **1**–**3** (DMSO-*d_6_*, *δ* in ppm).

Position	1			2		3	
	*δ*_C_, Type	*δ*_H_, (*J* in Hz)	*δ*_H_, (*J* in Hz) ^a^	*δ*_C_, Type	*δ*_H_, (*J* in Hz)	*δ*_C_, Type	*δ*_H_, (*J* in Hz)
2	144.8, C			144.7, C		144.7, C	
4	125.5, C			125.5, C		125.5, C	
5	125.5, C			125.5, C		125.5, C	
6	23.8, CH_2_	2.41, t (8.0)	2.20, m	23.8, CH_2_	2.40, t (7.8)	23.7, CH_2_	2.30, t (8.1)
7	20.6, CH_2_	1.34, m	1.25, m	20.6, CH_2_	1.34, m	20.5, CH_2_	1.29, m
8	13.3, CH_3_	0.80, t (7.3)	0.69, t (7.3)	13.3, CH_3_	0.80, t (7.2)	13.2, CH_3_	0.71, t (7.3)
9	7.9, CH_3_	2.15, s	2.03, s	7.9, CH_3_	2.13, s	8.0, CH_3_	2.22, s
10	8.0, CH_3_	2.20, s	2.10, s	8.0, CH_3_	2.19, s	8.0, CH_3_	2.22, s
1′ NH		11.11, s	10.80, s		11.02, s		11.03, s
2′	123.9, CH	7.17, s ^b^	7.02, s ^b^	123.8, CH	7.16, s	123.8, CH	7.16, s
3′	109.1, C			109.1, C		109.1, C	
4′	126.8, C			126.8, C		126.8, C	
5′	117.5, CH	7.39, d (7.3)	7.27, d (7.9)	117.5, CH	7.40, d (7.9)	117.5, CH	7.38, m
6′	118.6, CH	7.00, t (7.7)	6.93, d (7.5)	118.6, CH	7.00, t (7.4)	118.6, CH	7.01, t (7.5)
7′	121.2, CH	7.09, t (7.5)	7.02, m ^b^	121.2, CH	7.09, t (7.4)	121.2, CH	7.09, t (7.5)
8′	111.6, CH	7.38, d (7.6)	7.32, d (8.1)	111.6, CH	7.38, d (8.0)	111.6, CH	7.38, m
9′	136.1, C			136.1, C		136.1, C	
10′	25.0, CH_2_	3.08, t (7.0)	3.03, t (6.8)	25.0, CH_2_	3.07, t (6.7)	24.9, CH	2.95, t (7.2)
11′	45.8, CH_2_	4.30, t (7.0)	4.21, t (6.7)	45.7, CH_2_	4.29, t (6.7)	45.6, CH	4.23, t (7.3)
1″ ^c^	136.8, C			126.7, C			11.03, s
2″	128.9, CH	7.16, m ^b^	7.02, m ^b^	129.9, CH	6.91, d (7.9)	123.8, CH	7.16, s
3″	128.6, CH	7.32, t (7.3)	7.21, m	115.3, CH	6.70, d (8.0)	109.1, C	
4″	127.0, CH	7.28, m	7.17, m	156.5, C		126.8, C	
5″	128.6, CH	7.32, t (7.3)	7.21, m	115.3, CH	6.70, d (8.0)	117.5, CH	7.38, m
6″	128.9, CH	7.16, m ^b^	7.02, m ^b^	129.9, CH	6.91, d (7.9)	118.6, CH	7.01, t (7.5)
7″	35.0, CH_2_	2.80, t (7.5)	2.70, t (7.3)	34.2, CH_2_	2.69, t (6.9)	121.2, CH	7.09, t (7.5)
8″	45.8, CH_2_	4.22, t (7.5)	4.09, t (7.2)	46.2, CH_2_	4.14, t (6.9)	111.6, CH	7.38, m
9″						136.1, C	
10″						24.9, CH	2.95, t (7.2)
11″						45.6, CH	4.23, t (7.3)

**^a^** ^1^H NMR data of compound **1** with TFA added. **^b^** Signals overlapped. **^c^** NH at 1″ for compound **3**.

**Table 2:** ^1^H (700 MHz) and ^13^C (175 MHz) NMR data of compounds **4**–**6** (DMSO-*d_6_*, *δ* in ppm).

Position	4		5		6	
	*δ*_C_, Type	*δ*_H_, (*J* in Hz)	*δ*_C_, Type ^a^	*δ*_H_, (*J* in Hz)	*δ*_C_, Type	*δ*_H_, (*J* in Hz)
2	144.8, C		144.8, C		146.0, C	
4	125.5, C		130.5, C		125.5, C	
5	125.6, C		125.7, C		125.5, C	
6	23.9, CH_2_	2.53, t (7.9)	23.8, CH_2_	2.55, m	16.1, CH_2_	2.67, q (7.6)
7	20.7, CH_2_	1.40, m	20.4, CH_2_	1.43, m	11.8, CH_3_	1.02, t (7.6)
8	13.4, CH_3_	0.89, t (7.2)	13.1, CH_3_	0.90, t (7.2)	7.9, CH_3_	2.11, s
9	7.9, CH_3_	2.10 or 2.11, s	15.1, CH_2_	2.57, q (7.6)	7.9, CH_3_	2.11, s
10	7.9, CH_3_	2.10 or 2.11, s	13.3, CH_3_	1.07, td (7.5, 1.9)		
11			7.6, CH_3_	2.10, d (1.5)		
1′	136.9, C		136.8, C		136.9, C	
2′	129.0, CH	7.19, d (7.2)	128.7, CH	7.20 or 7.17, d (7.1)	129.0, CH	7.19, d (7.1)
3′	128.6, CH	7.33, t (7.3)	128.4, CH	7.33, m	128.6, CH	7.33, t (7.3)
4′	127.0, CH	7.28, t (7.2)	126.8, CH	7.29, m	127.0, CH	7.28, t (7.3)
5′	128.6, CH	7.33, t (7.3)	128.4, CH	7.33, m	128.6, CH	7.33, t (7.3)
6′	129.0, CH	7.19, d (7.2)	128.7, CH	7.20 or 7.17, d (7.1)	129.0, CH	7.19, d (7.1)
7′	35.0, CH_2_	2.93, t (7.0)	35.2, CH_2_	2.93, m	35.0, CH_2_	2.95, t (7.3)
8′	46.0, CH_2_	4.28, t (7.0)	45.6, CH_2_	4.29, m	45.9, CH_2_	4.28, t (7.3)
1″	126.5, C		126.4, C		136.9, C	
2″	129.9, CH	6.92, d (8.1)	129.6, CH	6.93 or 6.90, d (8.2)	129.0, CH	7.19, d (7.1)
3″	115.4, CH	6.70, d (8.1)	115.2, CH	6.71 or 6.70, d (8.4)	128.6, CH	7.33, t (7.3)
4″	156.8, C		156.7, C		127.0, CH	7.28, t (7.3)
5″	115.4, CH	6.70, d (8.1)	115.2, CH	6.71 or 6.70, d (8.4)	128.6, CH	7.33, t (7.3)
6″	129.9, CH	6.92, d (8.1)	129.6, CH	6.93 or 6.90, d (8.2)	129.0, CH	7.19, d (7.1)
7″	34.2, CH_2_	2.81, t (6.8)	34.3, CH_2_	2.81, m	35.0, CH_2_	2.95, t (7.3)
8″	46.4, CH_2_	4.21, t (6.9)	46.0, CH_2_	4.21, m	45.9, CH_2_	4.28, t (7.3)

**^a^** Deduced from HSQC and HMBC spectra.

**Table 3:** ^1^H (700 MHz) and ^13^C (175 MHz) NMR data of compound **7** (DMSO-*d_6_*, *δ* in ppm).

Position	*δ*_C_, Type	*δ*_H_, (*J* in Hz)	Position	*δ*_C_, Type	*δ*_H_, (*J* in Hz)
2	153.1, C		1′ NH		11.06, s
3	142.6, C		2′	124.2, CH	7.13, s
4	144.5, CH	8.20, s	3′	108.3, C	
5	140.6, C		4′	126.8, C	
6	142.6, CH	8.40, s	5′	117.3, CH	7.25, d (7.9)
7	29.5, CH_2_	2.83, t (8.2)	6′	118.6, CH	6.91, t (7.4)
8	21.8, CH_2_	1.56, m	7′	121.2, CH	7.06, t (7.3)
9	13.8, CH_3_	1.00, t (7.0)	8′	111.6, CH	7.35, d (8.1)
10	24.5, CH_2_	2.73, q (7.5)	9′	136.0, C	
11	14.4, CH_3_	1.13, t (7.5)	10′	26.5, CH_2_	3.35, m ^a^
12	24.5, CH_2_	2.57, q (7.5)	11′	58.5, CH_2_	4.78, t (6.5)
13	13.9, CH_3_	1.01, t (7.5)			

**^a^** Signal overlapped with H_2_O.

**Table 4:** MIC values (μg/mL) for Compounds **1**–**7**.

Test organism	MIC (μg/mL, *n* = 3)									
	1	2	3	4	5	6	7	Rifampicin	Tetracycline	Gentamicin
*B. subtilis* DSM10	8	>32	8	>32	32	32	>32	<0.031	2–4	0.06
*M. smegmatis* ATCC607	4	>32	4	>32	>32	16	>32	8–16	0.25–0.5	4–8 ^a^
*L. monocytogenes* DSM20600	32	>32	8	>32	>32	>32	>32	<0.031	0.5–1	<0.031
*S. aureus* ATCC25923	16	>32	8	>32	>32	32	>32	<0.031	0.25–0.5	0.06–0.125
*E. coli* ATCC25922	>32	>32	>32	>32	>32	>32	>32	4	2–4	0.06
								Nystatin	Tebuconazole	Amphotericin B
*C. albicans* FH2173 ^b^	>32	>32	16	>32	>32	>32	>32	1–2	0.25	0.5–1

**^a^** Isoniazid was used as positive control. **^b^** Nystatin, tebuconazole and amphotericin B were used as positive controls.

## Supplementary Materials

The supporting information is available free of charge at: https://www.mdpi.com/article/10.3390/md20100620/s1, Figures S1–S48: HR-ESI-MS, HR-ESI-MS/MS and NMR spectra for compounds 1–7; Figure S49: SDS-PAGE gel of the purified His-tagged DisA (PDF).
