# Supplementary material for: Seven New Alkaloids Isolated from Marine Flavobacterium Tenacibaculum discolor sv11
Source: Mar Drugs. 2022 Sep 30;20(10):620. doi: 10.3390/md20100620 (PMC9605681; doi:10.3390/md20100620)
Supplement: Supplementary file 1 [file marinedrugs-20-00620-s001.zip › marinedrugs-1925976-supplementary.pdf]

Supplementary Information

Seven New Alkaloids Isolated from Marine Flavobacterium *Tenacibaculum discolor* sv11

Lei Wang [1,2], Michael Marner [2], Ute Mettal [1,2], Yang Liu [1,2,*] and Till F. Schäberle [1,2,3,*]

1 Institute for Insect Biotechnology, Justus-Liebig-University Giessen, 35392 Giessen, Germany

2 Fraunhofer Institute for Molecular Biology and Applied Ecology (IME), Branch for Bioresources, 35392 Giessen, Germany

3 German Center for Infection Research (DZIF), Partner Site Giessen-Marburg-Langen, 35392 Giessen, Germany

* Correspondence: Till.F.Schaeberle@agrar.uni-giessen.de; Tel.: +49-(0)641-97219-140 (T.F.S.); Liu.Yang@agrar.uni-giessen.de (Y.L.)

Contents

Figure S1. The HR-ESI-MS and HR-ESI-MS/MS of compound 1...................................... 1

Figure S2. The ^{1}H-NMR (700 MHz, DMSO-d_6) spectrum of compound 1.......................... 2

Figure S3. The ^{13}C-NMR (175 MHz, DMSO-d_6) spectrum of compound 1......................... 2

Figure S4. The HSQC (700 MHz, DMSO-d_6) spectrum of compound 1............................. 3

Figure S5. The ^{1}H-^{1}H COSY (700 MHz, DMSO-d_6) spectrum of compound 1. 3

Figure S6. The ^{1}H-^{13}C HMBC (700 MHz, DMSO-d_6) spectrum of compound 1. 4

Figure S7. The ^{1}H-^{15}N HMBC (700 MHz, DMSO-d_6) spectrum of compound 1. 4

Figure S8. The ^{1}H-^{15}N HMBC (700 MHz, DMSO-d_6) spectrum of compound 1 (measured with non-uniform sampling). 5

Figure S9. The ^{1}H-NMR (700 MHz, DMSO-d_6) spectrum of compound 1 with TFA added. 5

Figure S10. The HSQC (700 MHz, DMSO-d_6) spectrum of compound 1 with TFA added. 6

Figure S11. The ^{1}H-^{1}H COSY (700 MHz, DMSO-d_6) spectrum of compound 1 with TFA added. 6

Figure S12. The ^{1}H-^{13}C HMBC (700 MHz, DMSO-d_6) spectrum of compound 1with TFA added. 7

Figure S13. The ^{1}H-^{15}N HMBC (700 MHz, DMSO-d_6) spectrum of compound 1 with TFA added. 7

Figure S14. The HR-ESI-MS and HR-ESI-MS/MS of compound 2.................................... 8

Figure S15. The ^{1}H-NMR (700 MHz, DMSO-d_6) spectrum of compound 2....................... 9

Figure S16. The ^{13}C-NMR (175 MHz, DMSO-d_6) spectrum of compound 2. 9

Figure S17. The HSQC (700 MHz, DMSO-d_6) spectrum of compound 2. 10

Figure S18. The ^{1}H-^{1}H COSY (700 MHz, DMSO-d_6) spectrum of compound 2. 10

Figure S19. The ^{1}H-^{13}C HMBC (700 MHz, DMSO-d_6) spectrum of compound 2. 11

Figure S20. The HR-ESI-MS and HR-ESI-MS/MS of compound 3.................................. 12

Figure S21. The ^{1}H-NMR (700 MHz, DMSO-d_6) spectrum of compound 3...................... 13

Figure S22. The ^{13}C-NMR (175 MHz, DMSO-d_6) spectrum of compound 3..................... 13

Figure S23. The HSQC (700 MHz, DMSO-d_6) spectrum of compound 3. 14

Figure S24. The ^{1}H-^{1}H COSY (700 MHz, DMSO-d_6) spectrum of compound 3. 14

Figure S25. The ^{1}H-^{13}C HMBC (700 MHz, DMSO-d_6) spectrum of compound 3. 15

Figure S26. The HR-ESI-MS and HR-ESI-MS/MS of compound 4.................................. 16

Figure S27. The ^{1}H-NMR (700 MHz, DMSO-d_6) spectrum of compound 4 17
Figure S28. The ^{13}C-NMR (175 MHz, DMSO-d_6) spectrum of compound 4 17
Figure S29. The HSQC (700 MHz, DMSO-d_6) spectrum of compound 4. 18
Figure S30. The ^{1}H-^{1}H COSY (700 MHz, DMSO-d_6) spectrum of compound 4. 18
Figure S31. The ^{1}H-^{13}C HMBC (700 MHz, DMSO-d_6) spectrum of compound 4. 19
Figure S32. The HR-ESI-MS and HR-ESI-MS/MS of compound 5.................................. 20
Figure S33. The ^{1}H-NMR (700 MHz, DMSO-d_6) spectrum of compound 5 21
Figure S34. The HSQC (700 MHz, DMSO-d_6) spectrum of compound 5. 21
Figure S35. The ^{1}H-^{1}H COSY (700 MHz, DMSO-d_6) spectrum of compound 5. 22
Figure S36. The ^{1}H-^{13}C HMBC (700 MHz, DMSO-d_6) spectrum of compound 5. 22
Figure S37. The HR-ESI-MS and HR-ESI-MS/MS of compound 6.................................. 23
Figure S38. The ^{1}H-NMR (700 MHz, DMSO-d_6) spectrum of compound 6 24
Figure S39. The ^{13}C-NMR (175 MHz, DMSO-d_6) spectrum of compound 6 24
Figure S40. The HSQC (700 MHz, DMSO-d_6) spectrum of compound 6. 25
Figure S41. The ^{1}H-^{1}H COSY (700 MHz, DMSO-d_6) spectrum of compound 6. 25
Figure S42. The ^{1}H-^{13}C HMBC (700 MHz, DMSO-d_6) spectrum of compound 6. 26
Figure S43. The HR-ESI-MS and HR-ESI-MS/MS of compound 7.................................. 27
Figure S44. The ^{1}H-NMR (700 MHz, DMSO-d_6) spectrum of compound 7 28
Figure S45. The ^{13}C-NMR (175 MHz, DMSO-d_6) spectrum of compound 7 28
Figure S46. The HSQC (700 MHz, DMSO-d_6) spectrum of compound 7. 29
Figure S47. The ^{1}H-^{1}H COSY (700 MHz, DMSO-d_6) spectrum of compound 7. 29
Figure S48. The ^{1}H-^{13}C HMBC (700 MHz, DMSO-d_6) spectrum of compound 7. 30
Figure S49. SDS-PAGE gel showing the purification of His-tagged DisA. 30

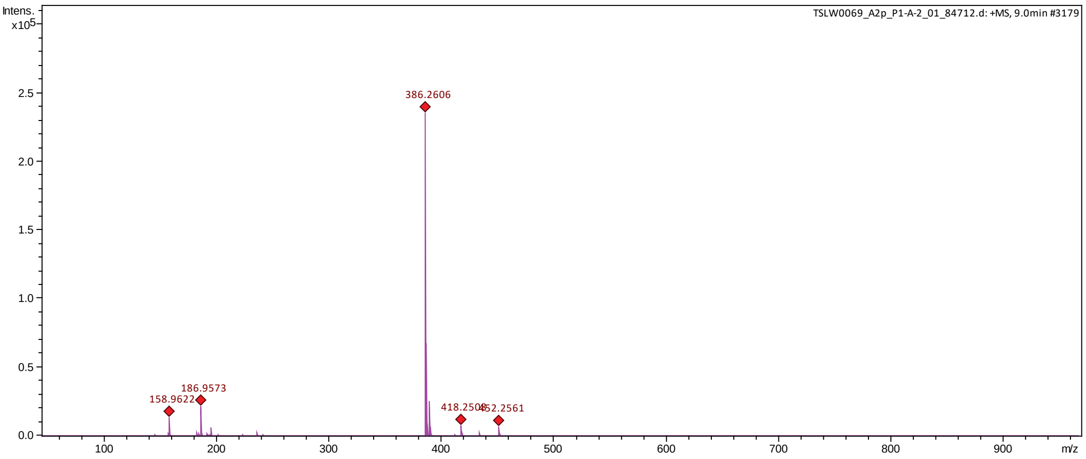

Meas. m/z	#	Ion Formula	m/z	err [ppm]	mSigma	# mSigma	Score	rdb	eÂ¯Conf	N-Rule
386.2606	1	C26H32N3	386.2591	-4.0	5.4	1	100.00	12.5	even	ok

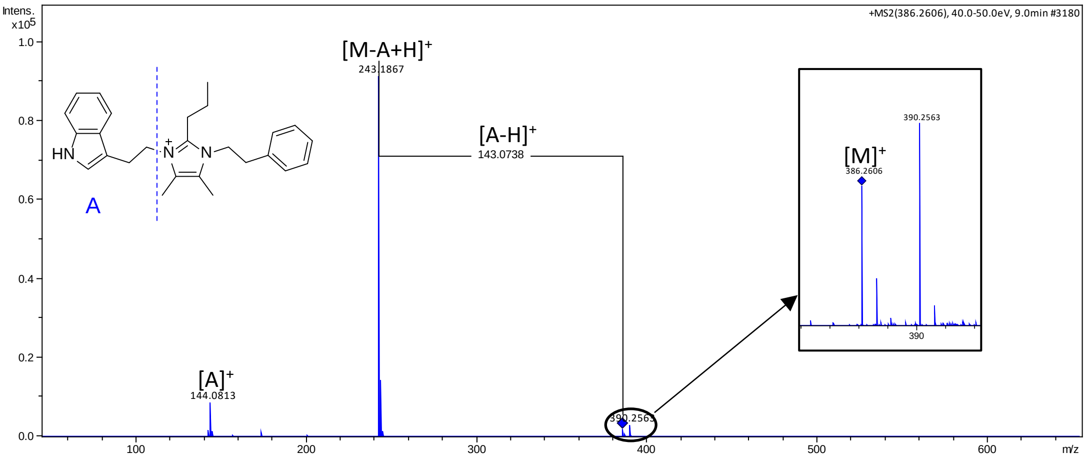

Figure S1. The HR-ESI-MS and HR-ESI-MS/MS of compound 1.

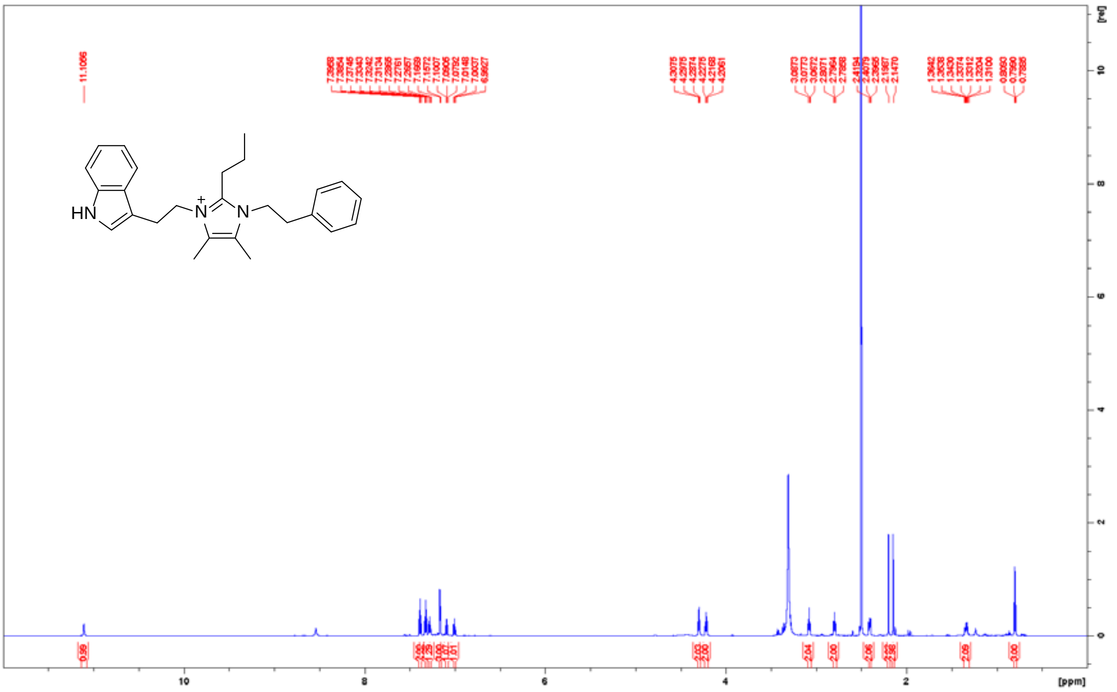

Figure S2. The ^{1}H-NMR (700 MHz, DMSO-d_6) spectrum of compound 1.

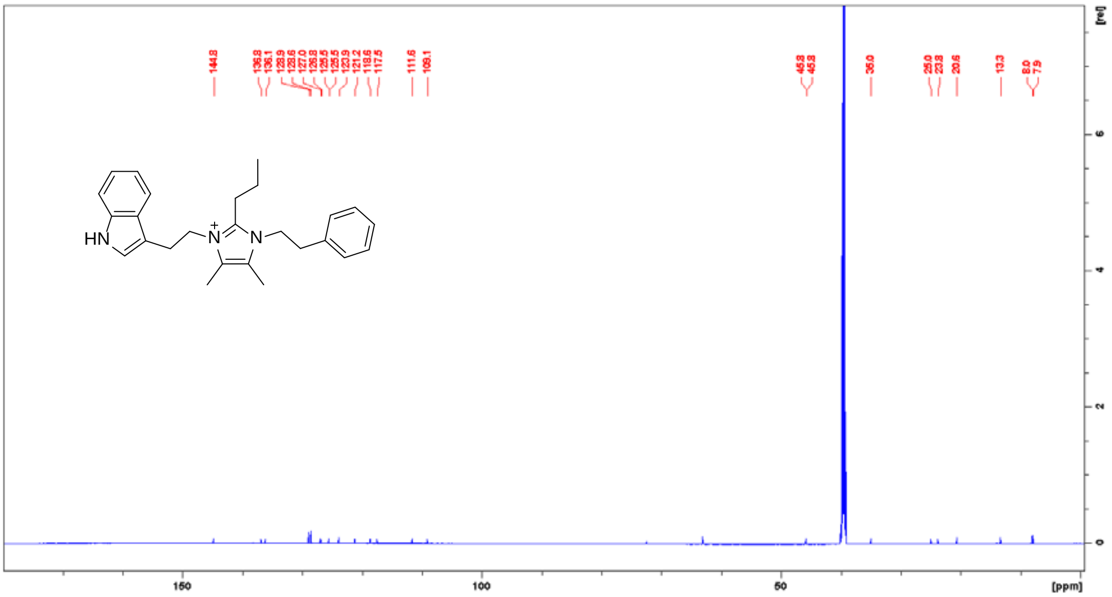

Figure S3. The ^{13}C-NMR (175 MHz, DMSO-d_6) spectrum of compound 1.

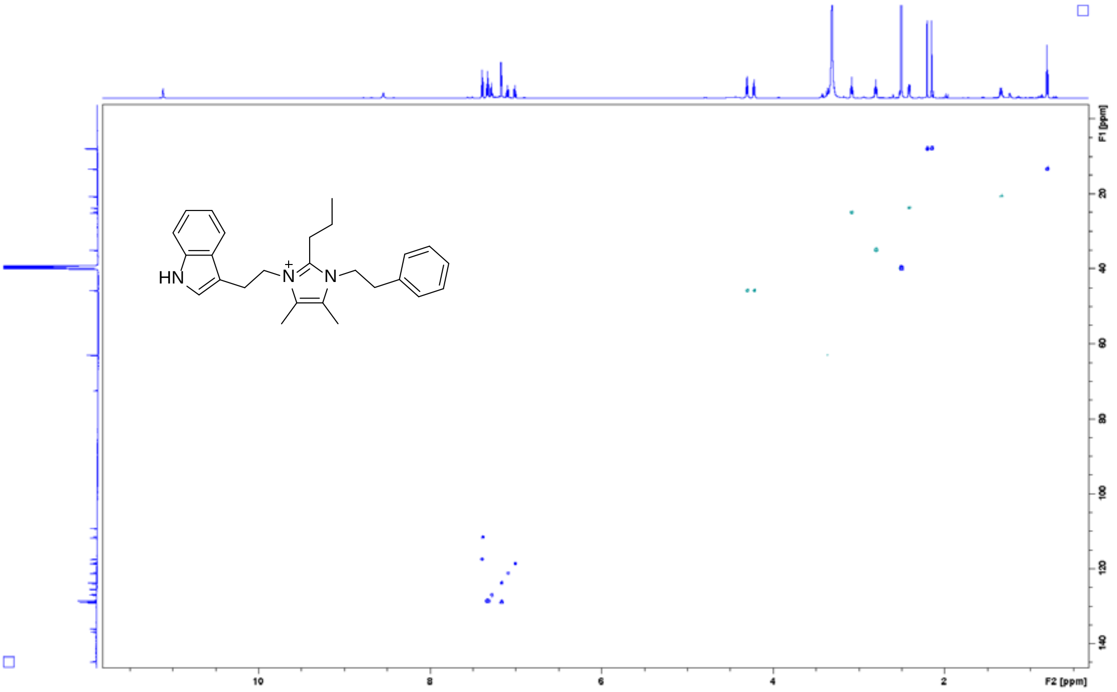

Figure S4. The HSQC (700 MHz, DMSO-d_6) spectrum of compound 1.

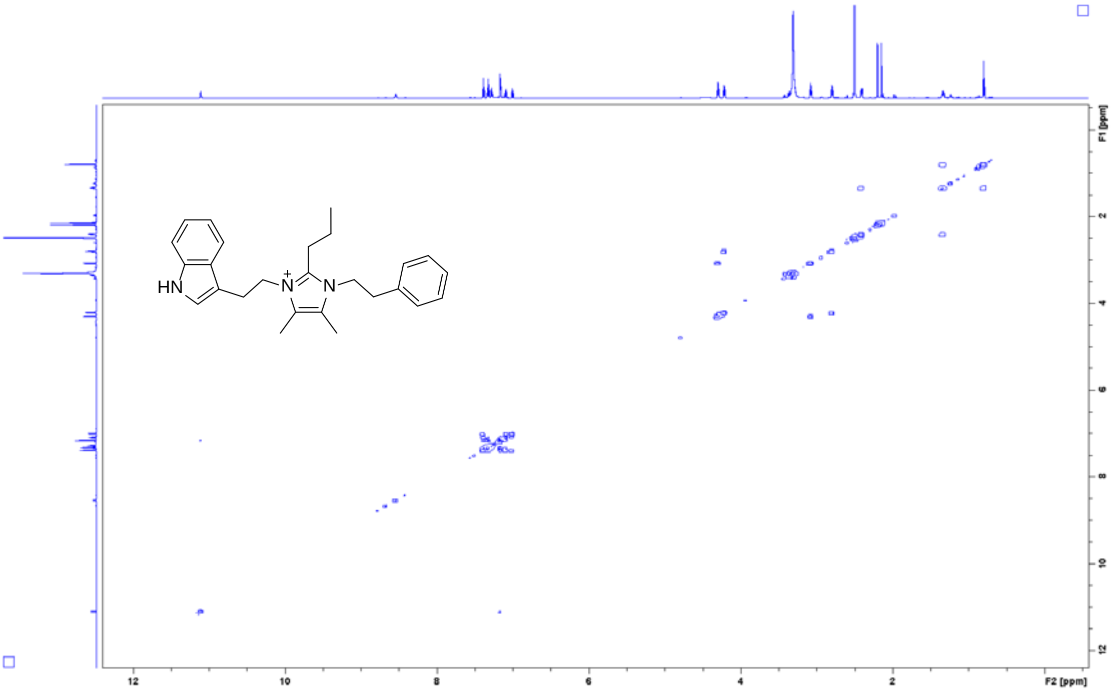

Figure S5. The ^{1}H-^{1}H COSY (700 MHz, DMSO-d_6) spectrum of compound 1.

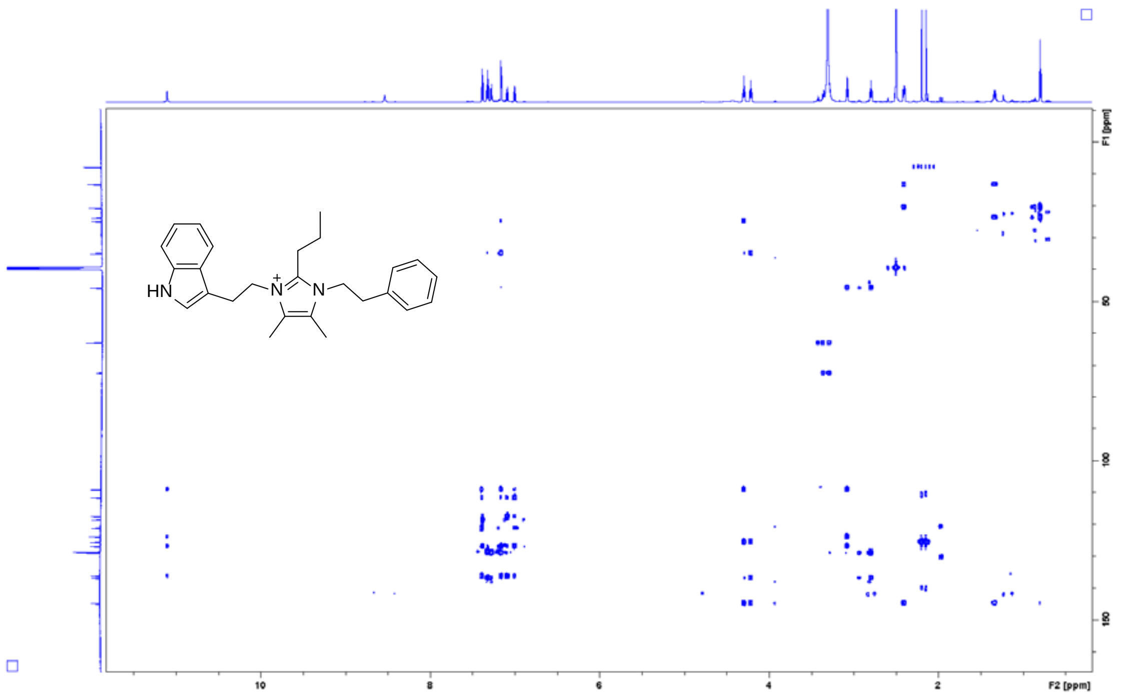

Figure S6. The ^{1}H-^{13}C HMBC (700 MHz, DMSO-d_6) spectrum of compound 1.

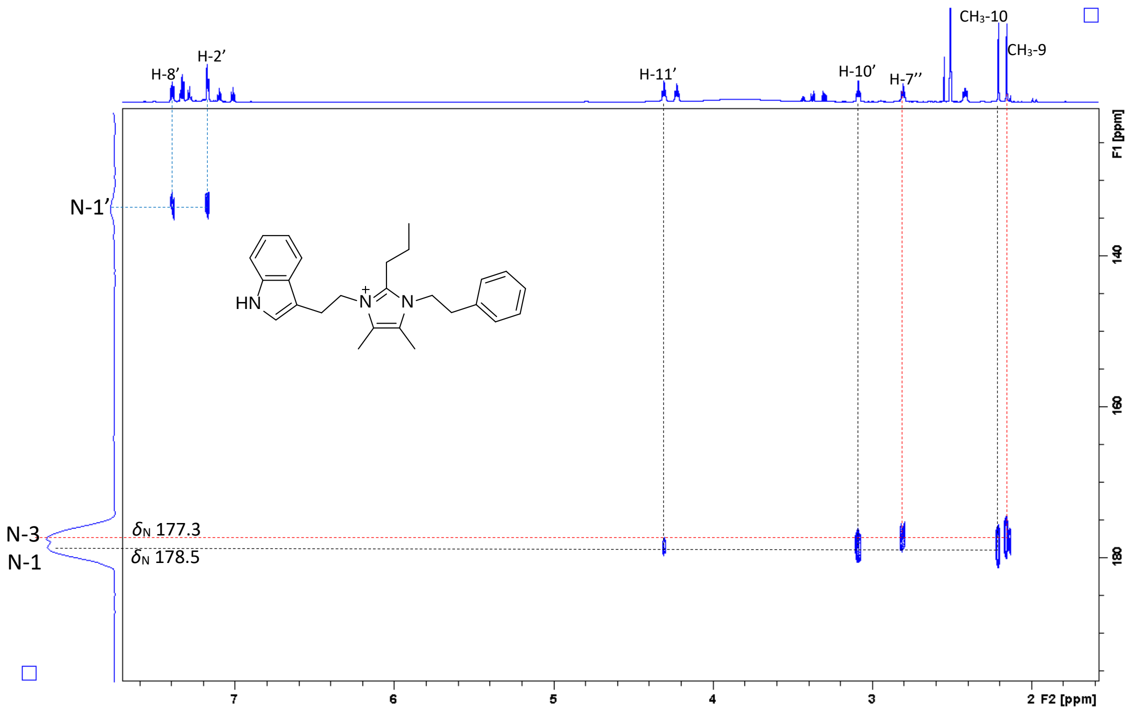

Figure S7. The ^{1}H-^{15}N HMBC (700 MHz, DMSO-d_6) spectrum of compound 1.

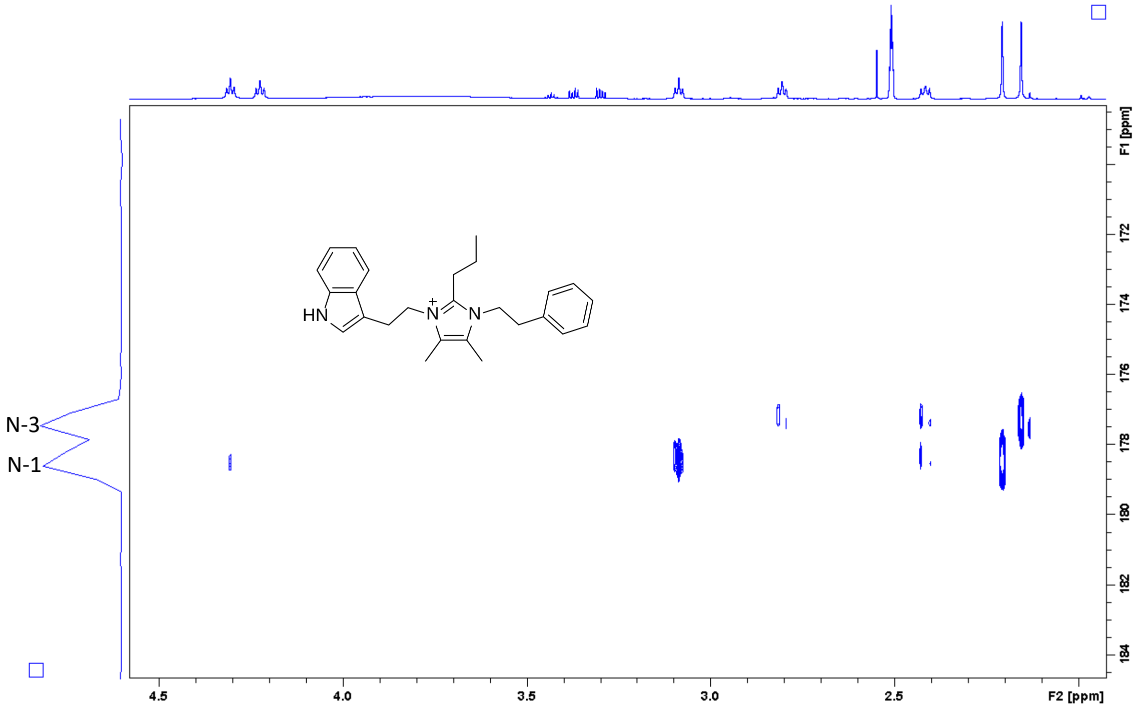

Figure S8. The ^{1}H-^{15}N HMBC (700 MHz, DMSO-d_6) spectrum of compound 1 (measured with non-uniform sampling).

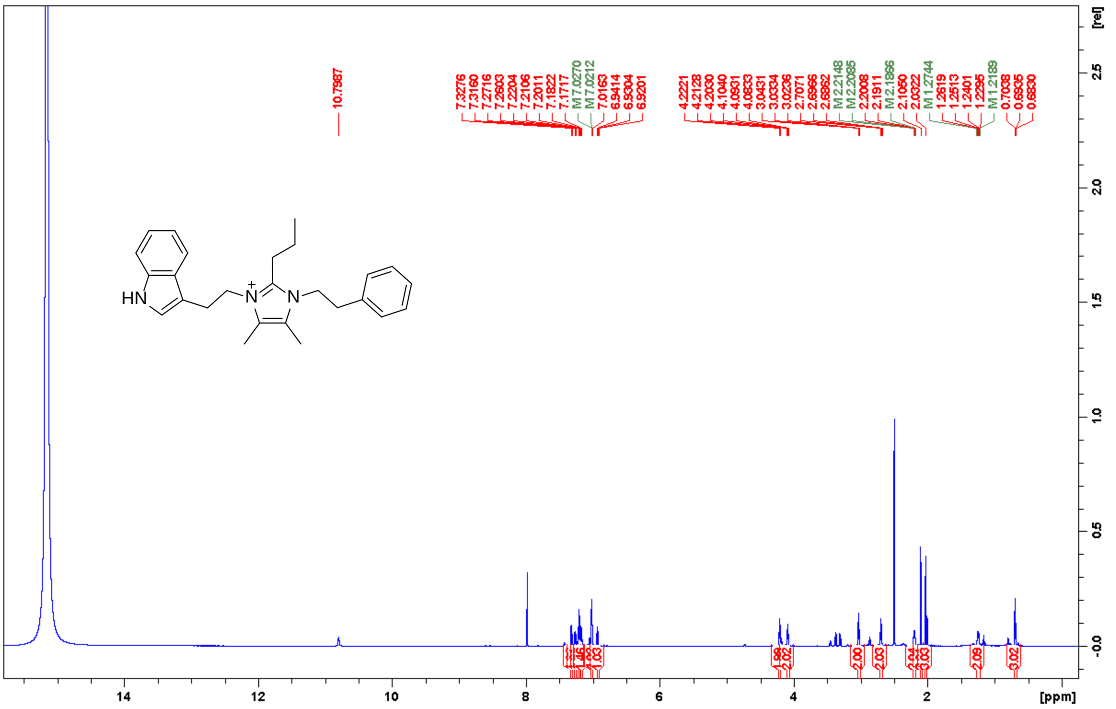

Figure S9. The ^{1}H-NMR (700 MHz, DMSO-d_6) spectrum of compound 1 with TFA added.

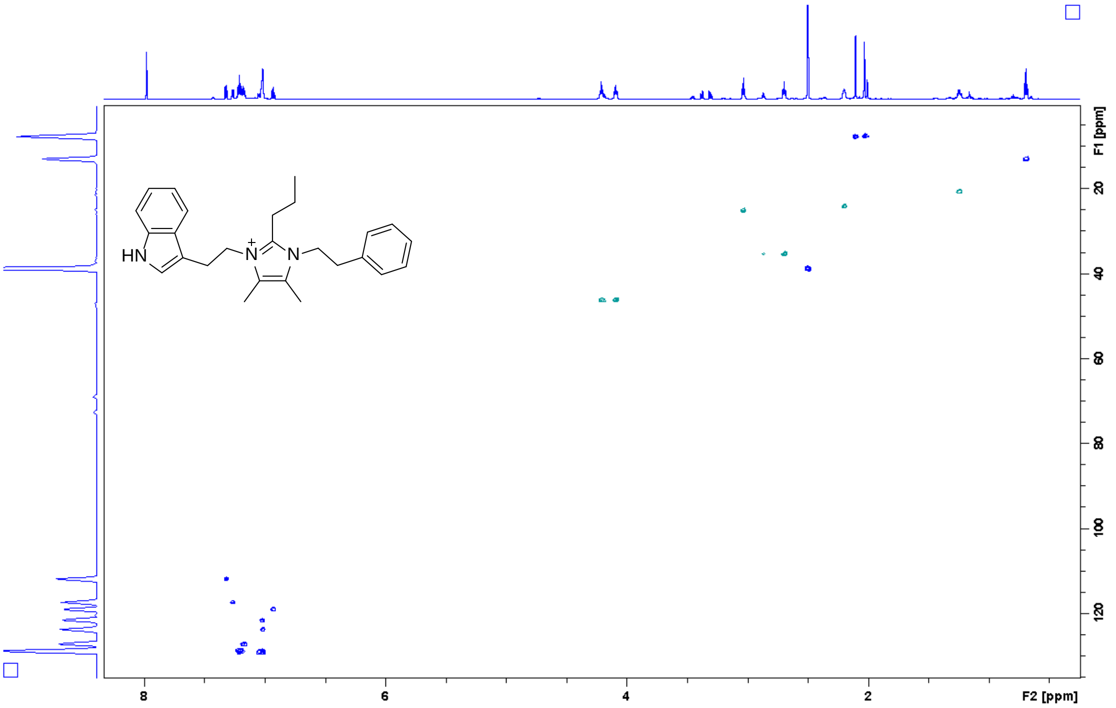

Figure S10. The HSQC (700 MHz, DMSO-d_6) spectrum of compound 1 with TFA added.

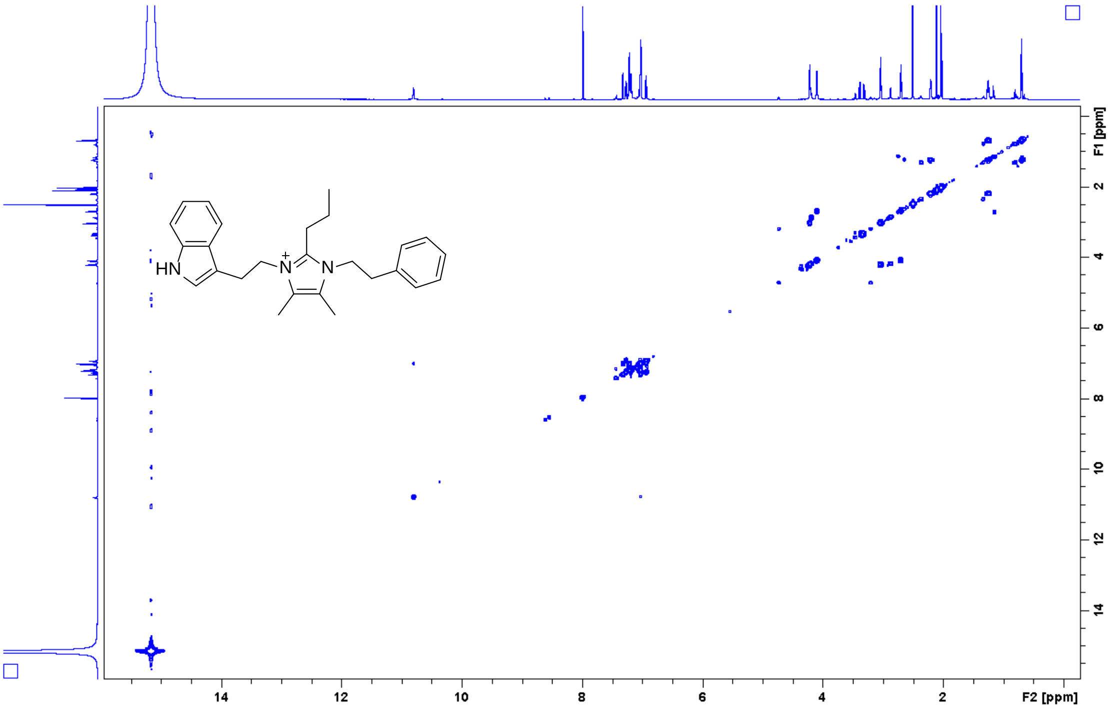

Figure S11. The ^{1}H-^{1}H COSY (700 MHz, DMSO-d_6) spectrum of compound 1 with TFA added.

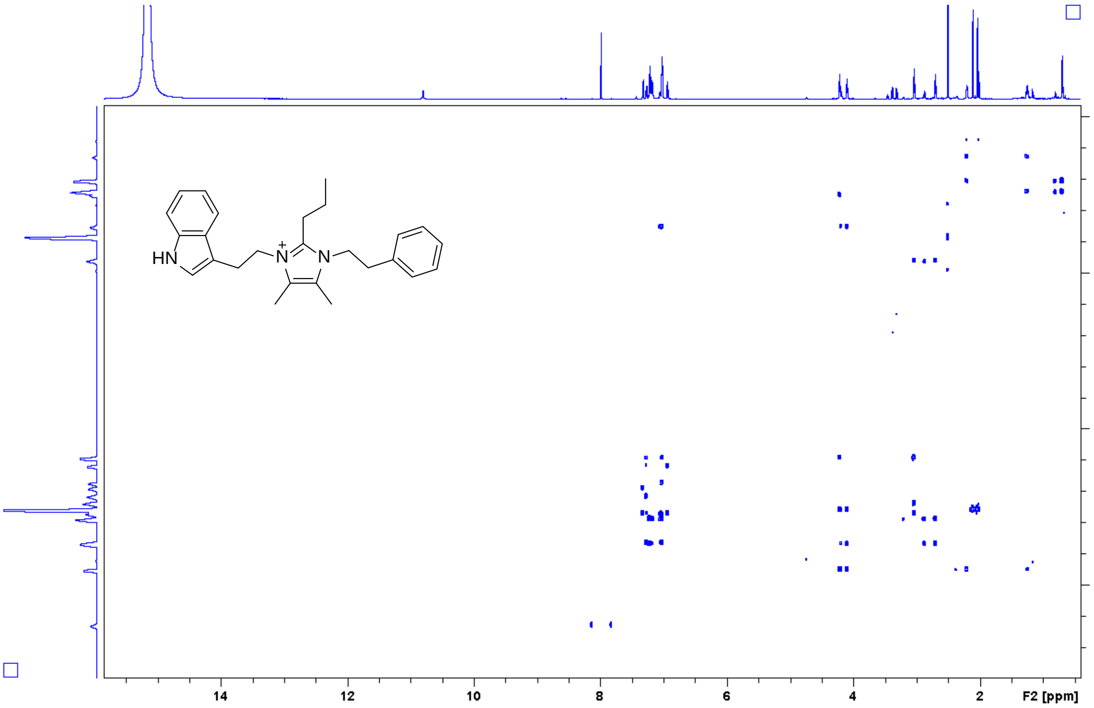

Figure S12. The ^{1}H-^{13}C HMBC (700 MHz, DMSO-d_6) spectrum of compound 1with TFA added.

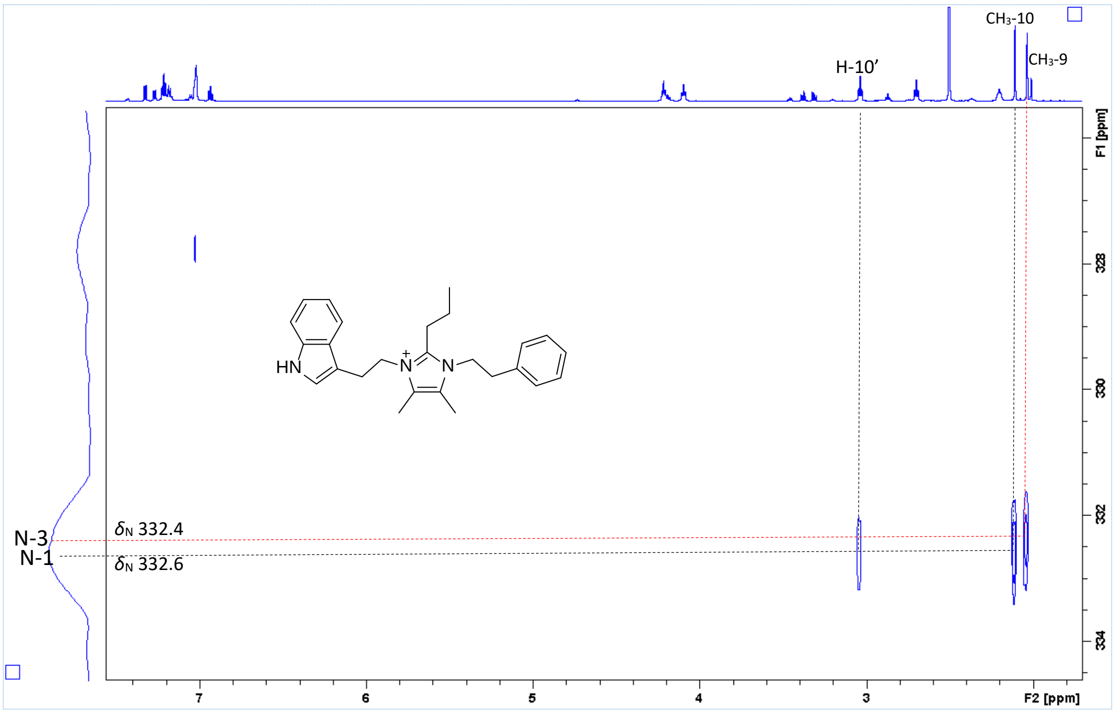

Figure S13. The ^{1}H-^{15}N HMBC (700 MHz, DMSO-d_6) spectrum of compound 1 with TFA added.

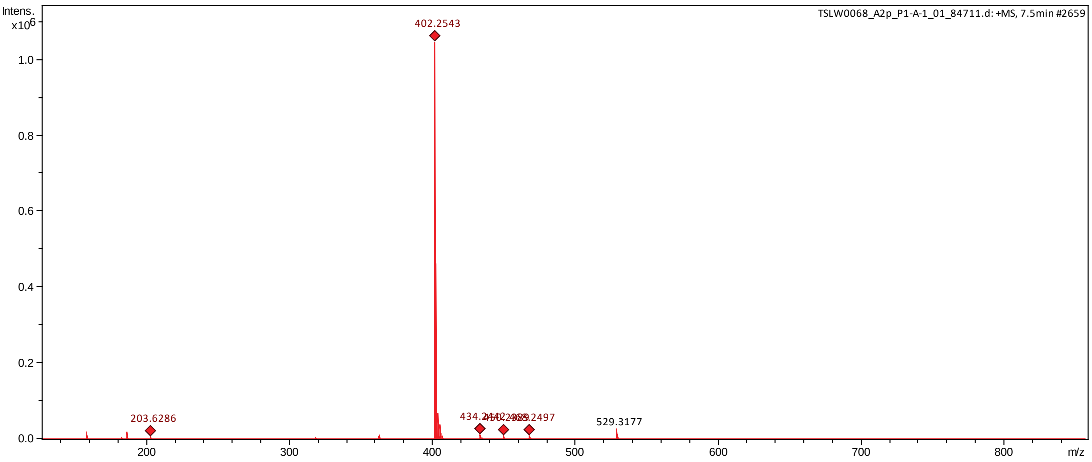

Meas. m/z	#	Ion Formula	m/z	err [ppm]	mSigma	# mSigma	Score	rdb	eÂ¯ Conf	N-Rule
402.2543	1	C26H32N3O	402.2540	-0.9	85.1	1	100.00	12.5	even	ok
402.2543	2	C14H36N5O8	402.2558	3.7	154.4	2	1.20	-0.5	even	ok
402.2543	3	C11H28N15O2	402.2545	0.4	154.9	3	2.40	5.5	even	ok

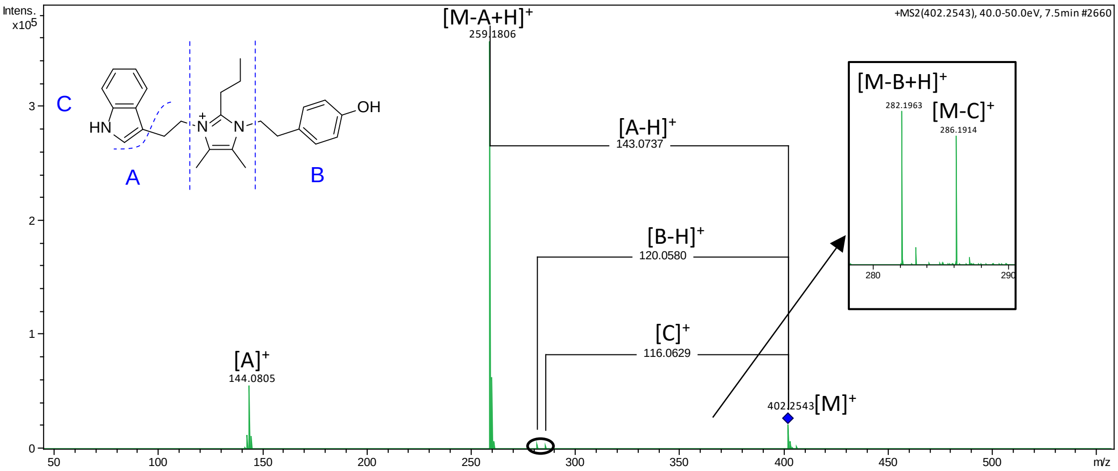

Figure S14. The HR-ESI-MS and HR-ESI-MS/MS of compound 2.

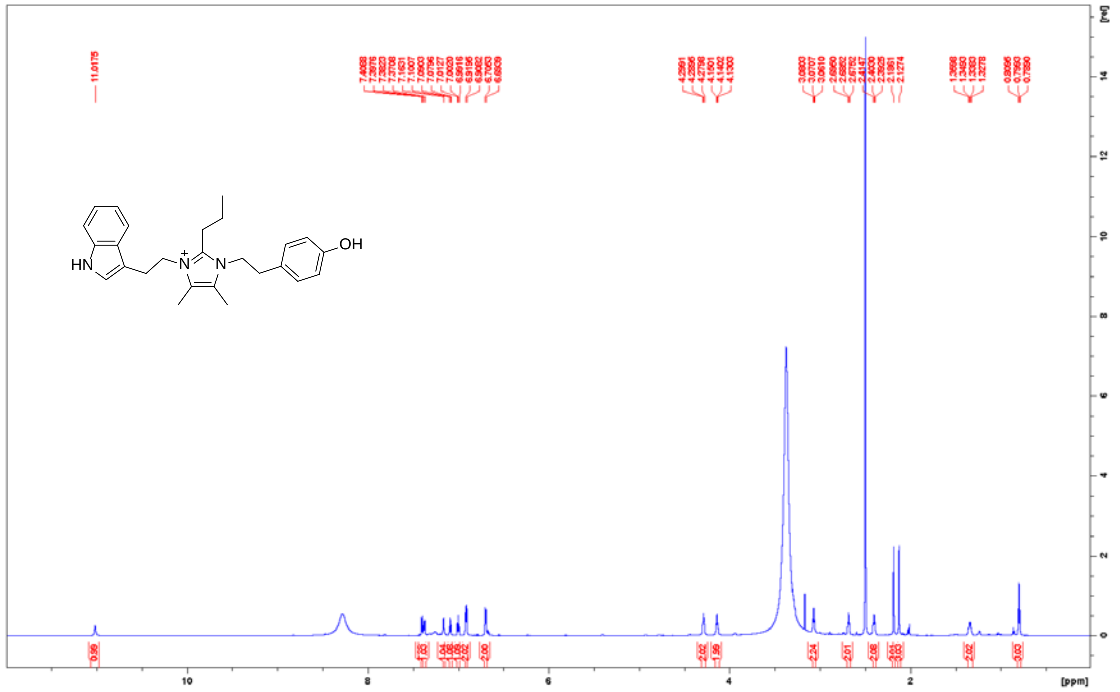

Figure S15. The ^{1}H-NMR (700 MHz, DMSO-d_6) spectrum of compound 2

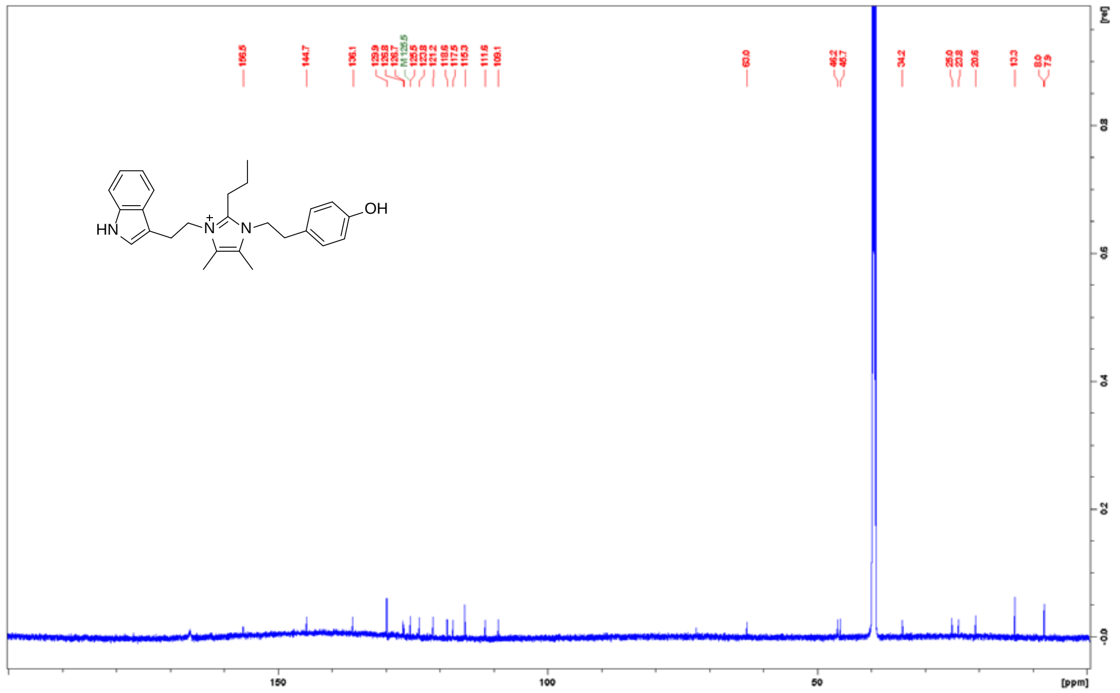

Figure S16. The ^{13}C-NMR (175 MHz, DMSO-d_6) spectrum of compound 2.

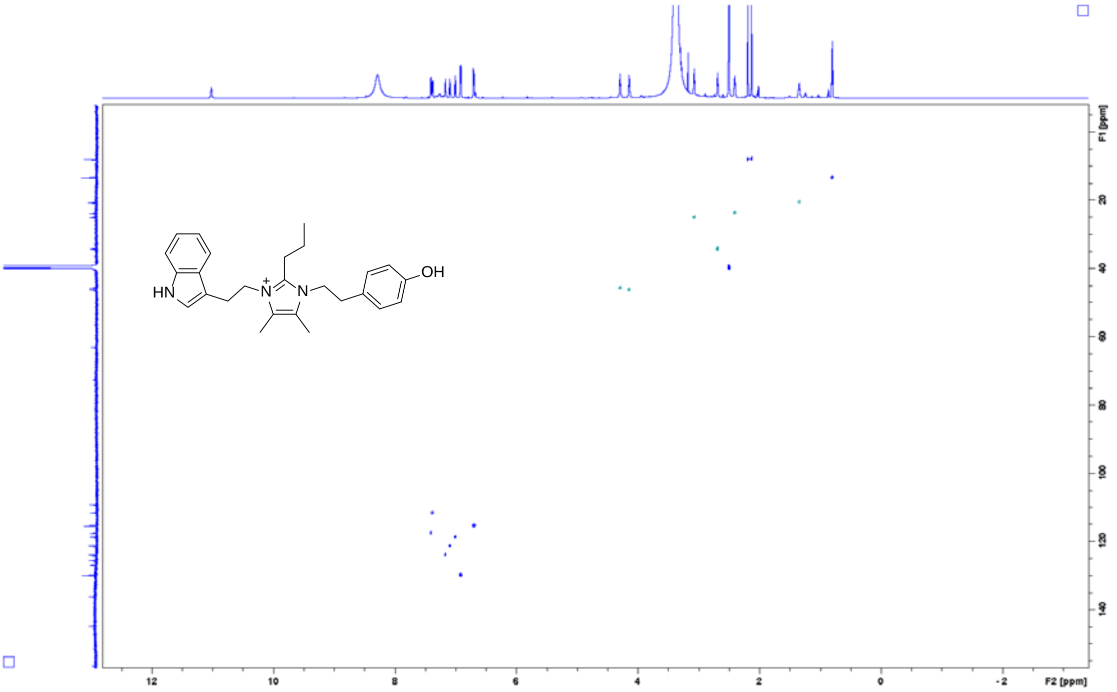

Figure S17. The HSQC (700 MHz, DMSO-d_6) spectrum of compound 2.

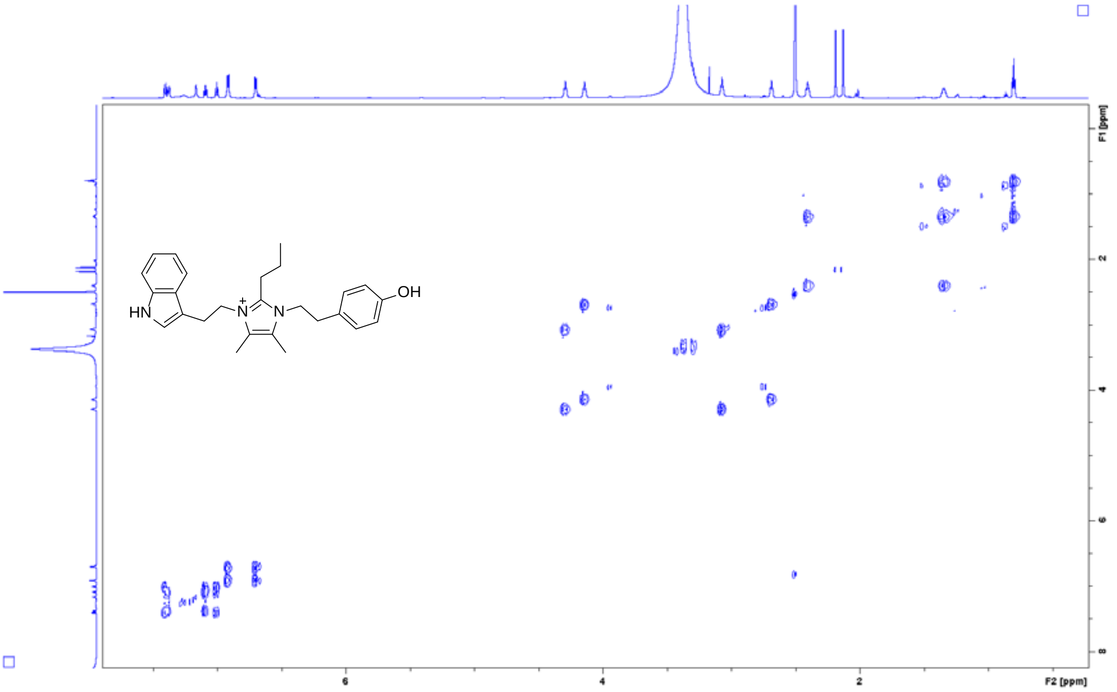

Figure S18. The ^{1}H-^{1}H COSY (700 MHz, DMSO-d_6) spectrum of compound 2.

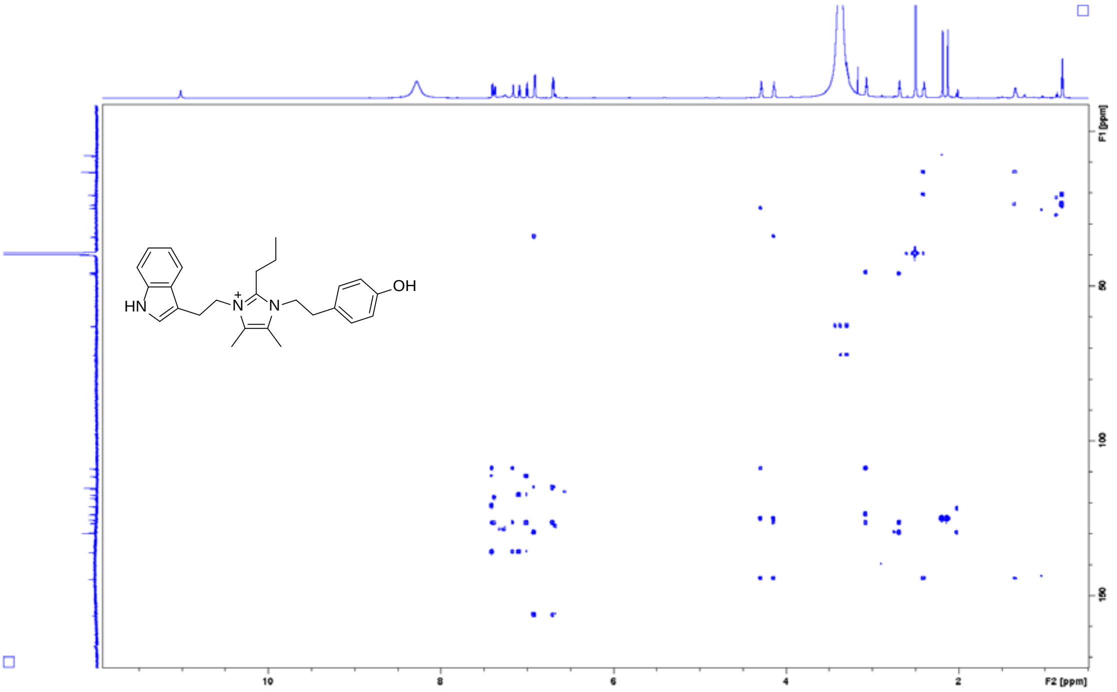

Figure S19. The ^{1}H-^{13}C HMBC (700 MHz, DMSO-d_6) spectrum of compound 2.

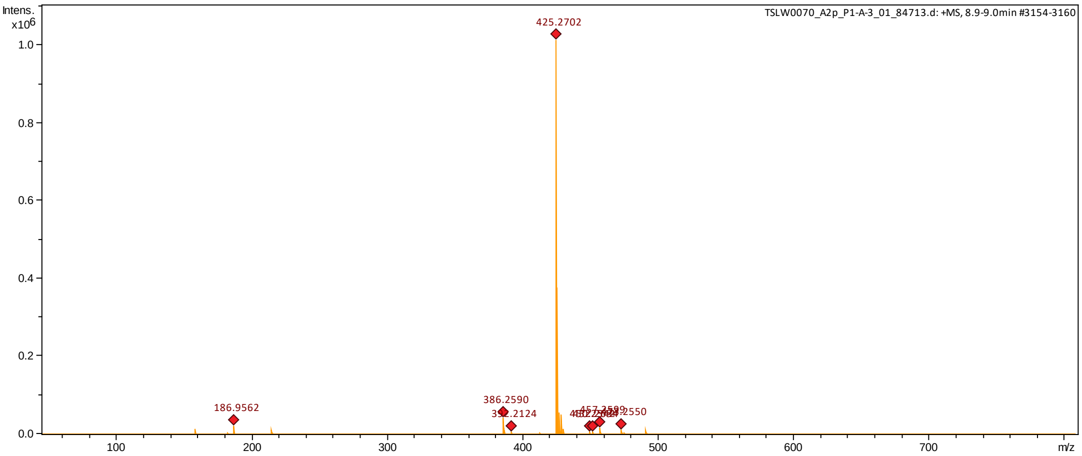

Meas. m/z	#	Ion Formula	m/z	err [ppm]	mSigma	# mSigma	Score	rdb	eÂ¯ Conf	N-Rule
425.2702	1	C28H33N4	425.2700	-0.6	29.0	1	100.00	14.5	even	ok
425.2702	2	C27H37O4	425.2686	-3.7	42.4	2	33.46	9.5	even	ok

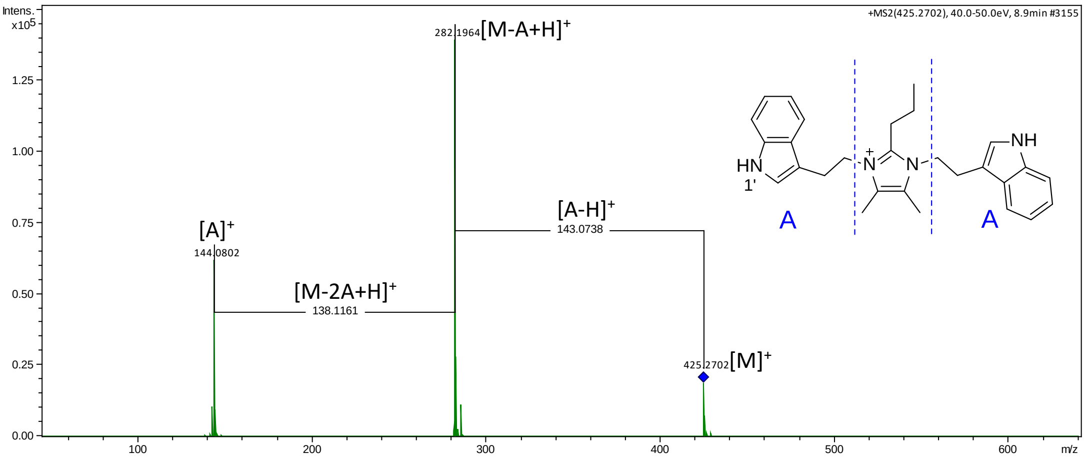

Figure S20. The HR-ESI-MS and HR-ESI-MS/MS of compound 3.

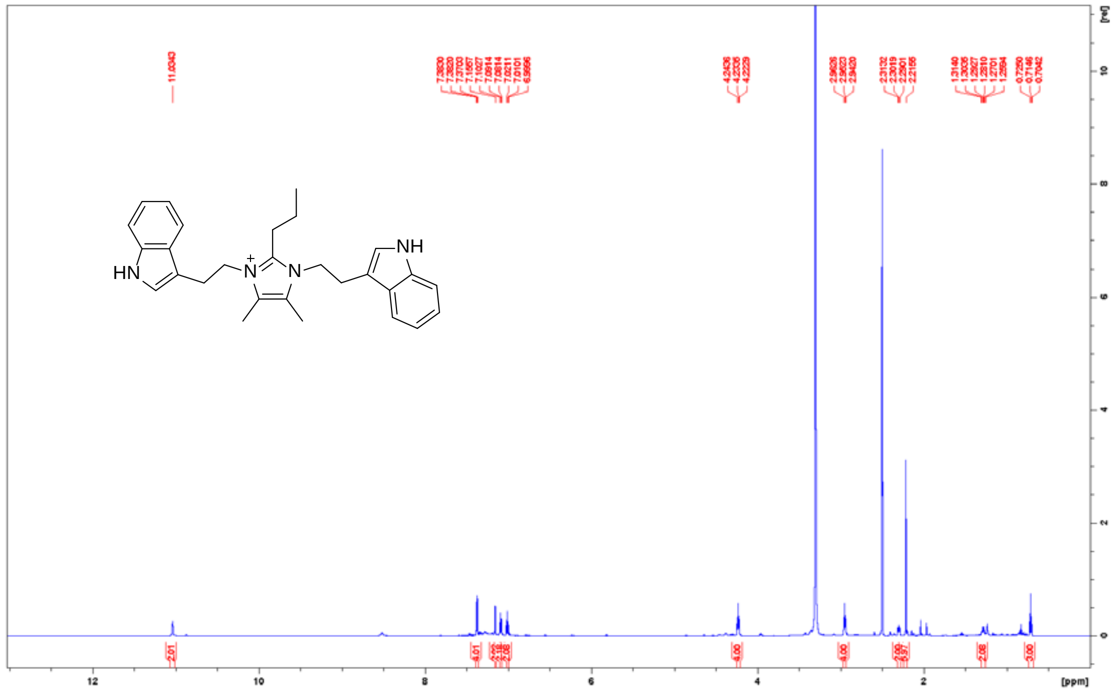

Figure S21. The ^{1}H-NMR (700 MHz, DMSO-d_6) spectrum of compound 3

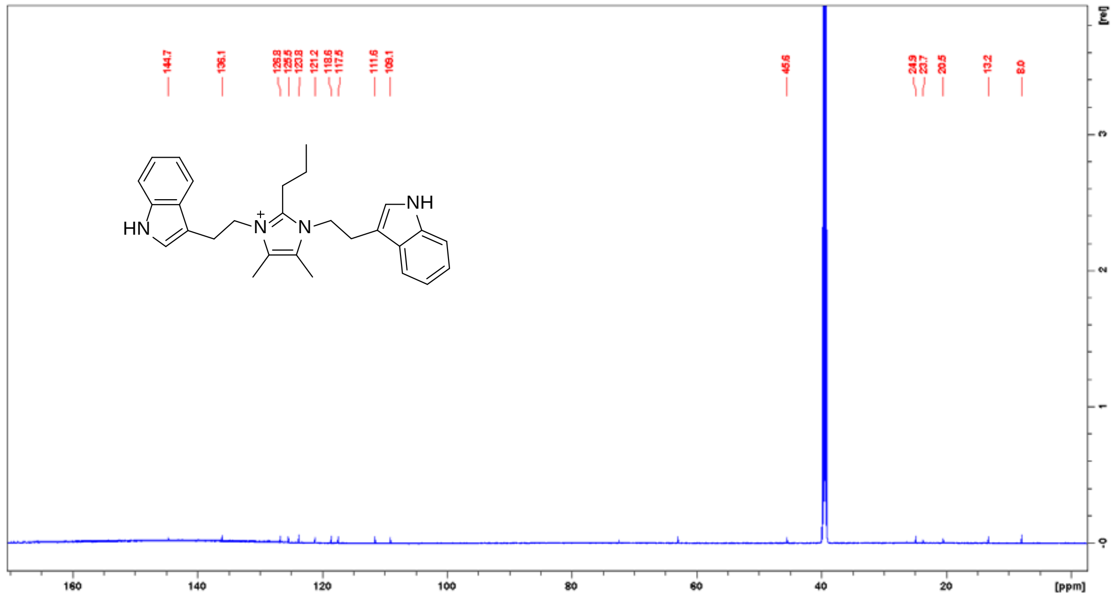

Figure S22. The ^{13}C-NMR (175 MHz, DMSO-d_6) spectrum of compound 3

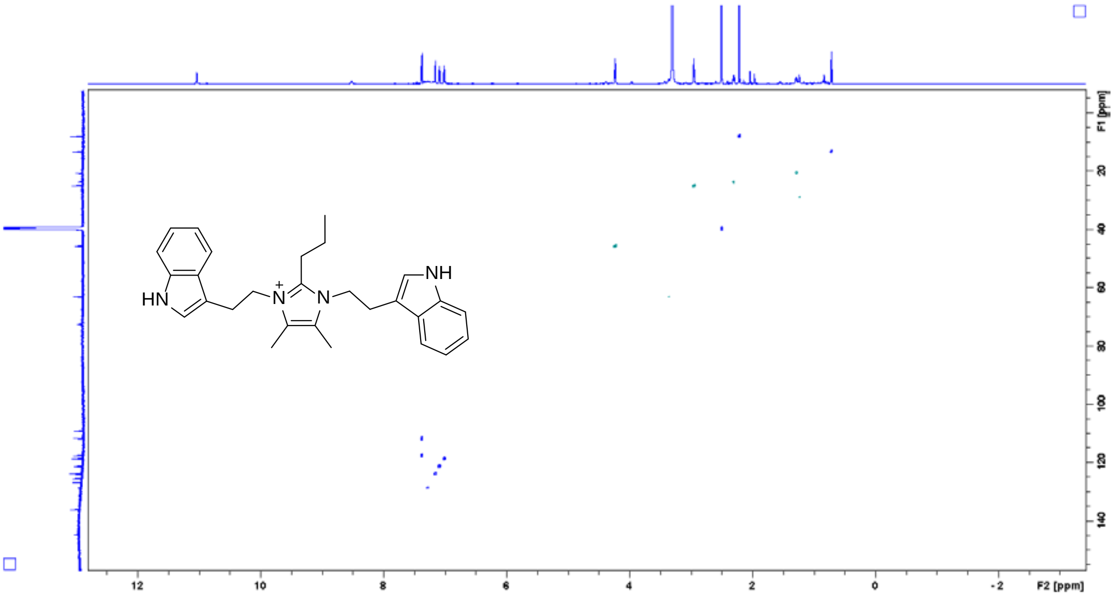

Figure S23. The HSQC (700 MHz, DMSO-d_6) spectrum of compound 3.

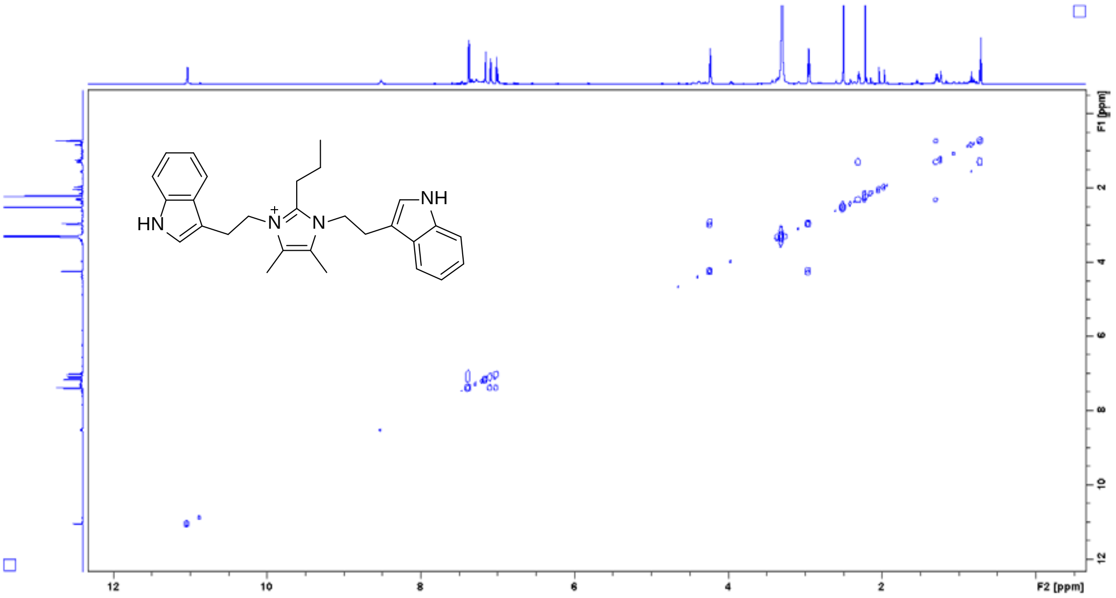

Figure S24. The ^{1}H-^{1}H COSY (700 MHz, DMSO-d_6) spectrum of compound 3.

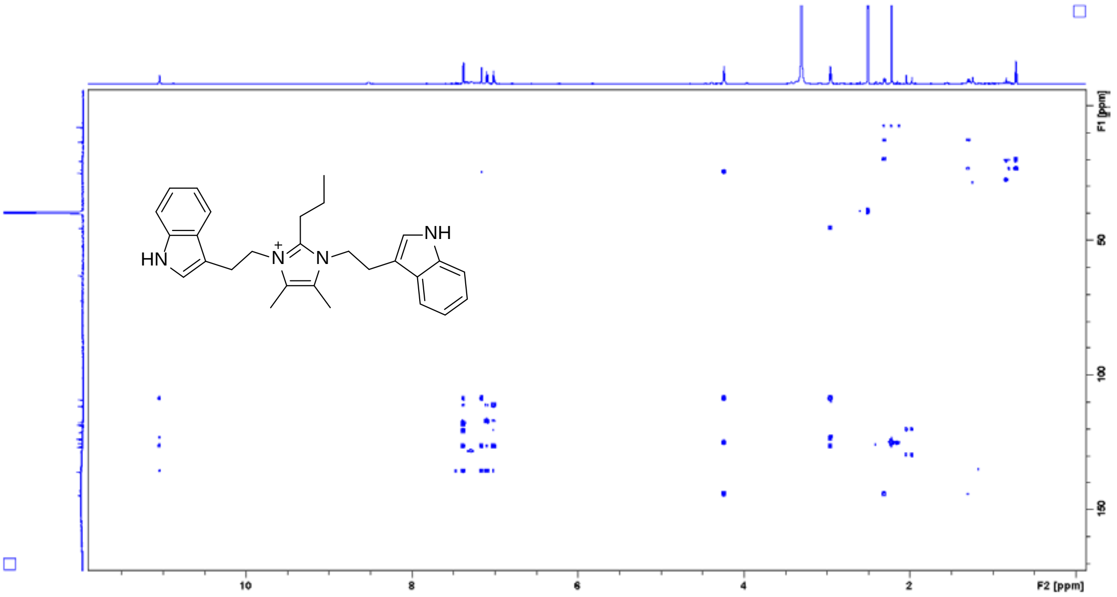

Figure S25. The ^{1}H-^{13}C HMBC (700 MHz, DMSO-d_6) spectrum of compound 3.

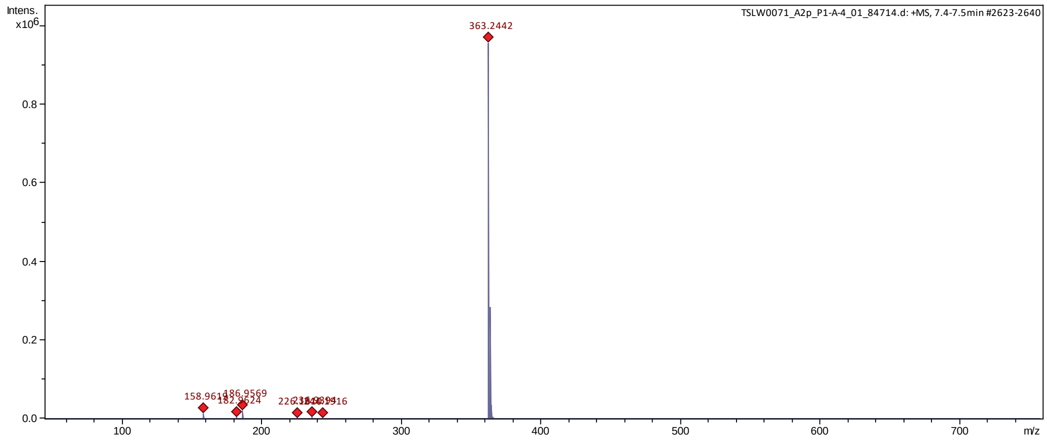

Meas. m/z	#	Ion Formula	m/z	err [ppm]	mSigma	# mSigma	Score	rdb	eÂ¯ Conf	N-Rule
363.2442	1	C24H31N2O	363.2431	-2.9	15.9	1	100.00	10.5	even	ok

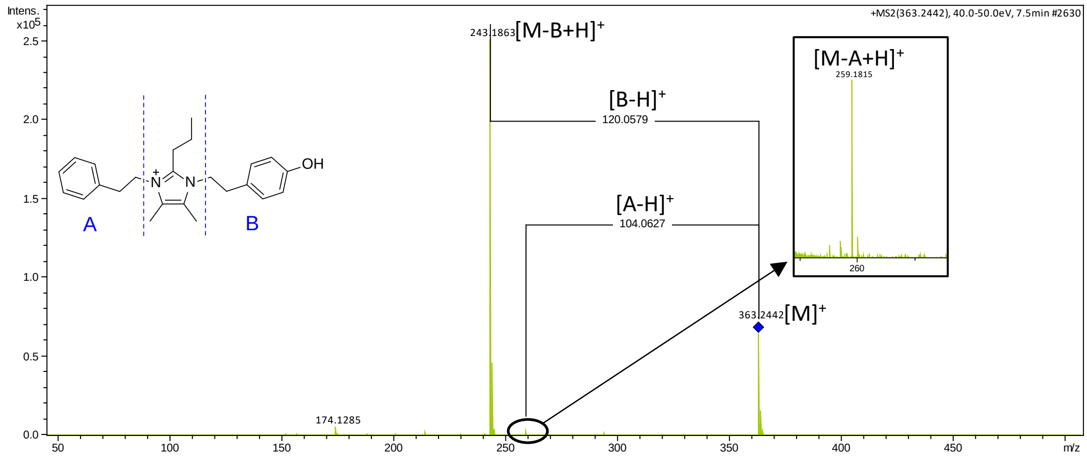

Figure S26. The HR-ESI-MS and HR-ESI-MS/MS of compound 4.

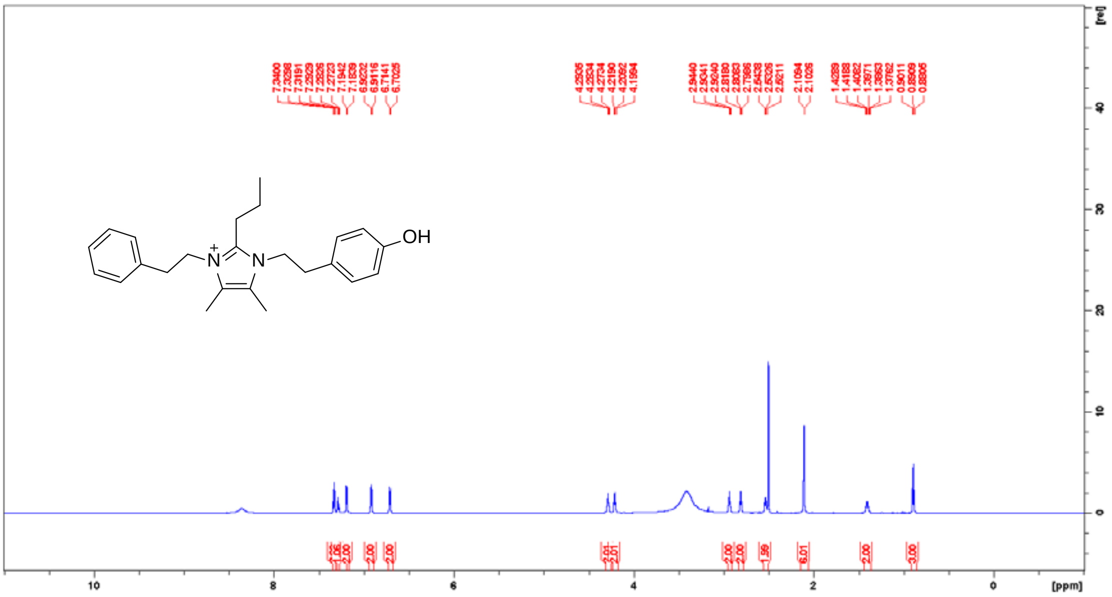

Figure S27. The ^{1}H-NMR (700 MHz, DMSO-d_6) spectrum of compound 4

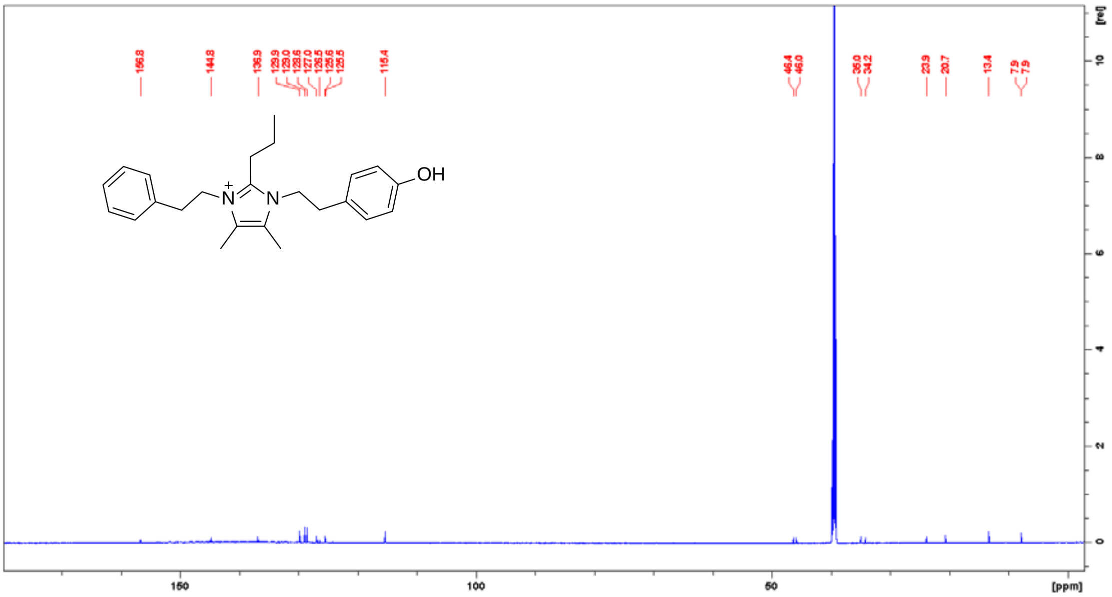

Figure S28. The ^{13}C-NMR (175 MHz, DMSO-d_6) spectrum of compound 4

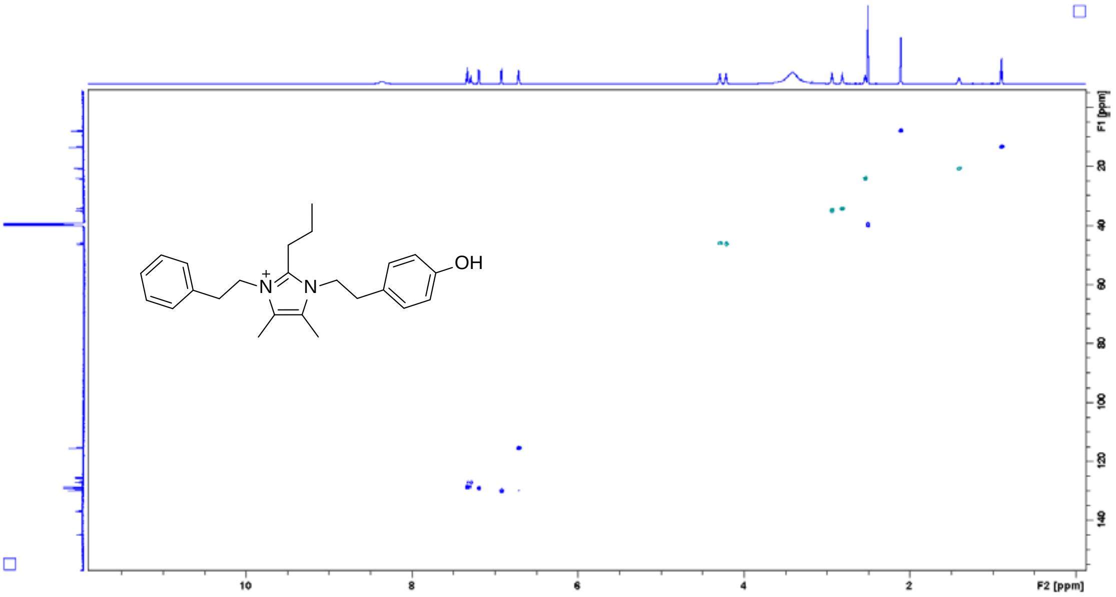

Figure S29. The HSQC (700 MHz, DMSO-d_6) spectrum of compound 4.

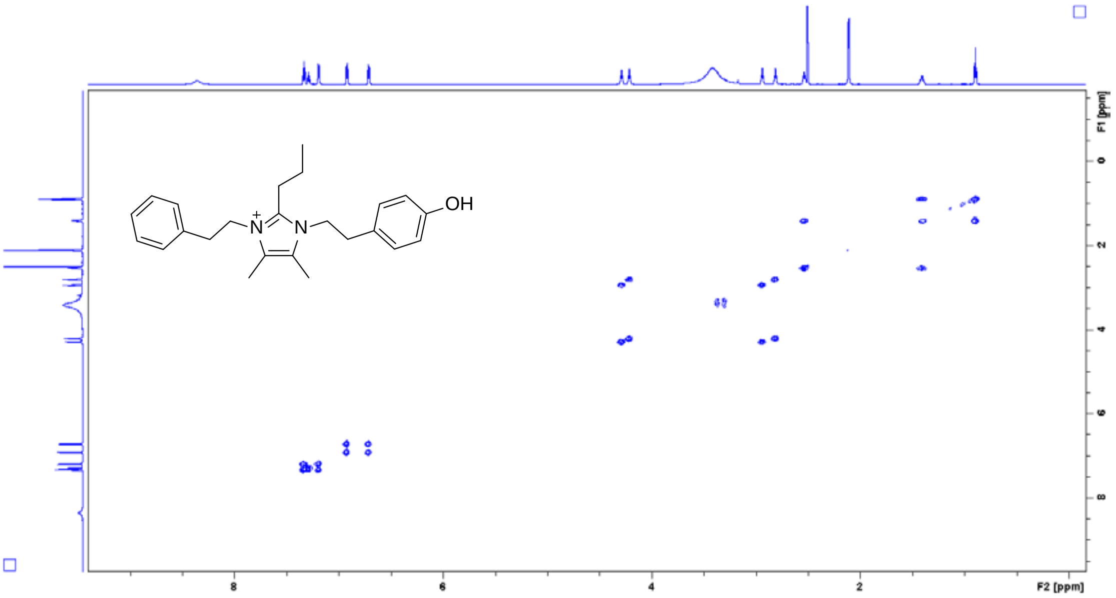

Figure S30. The ^{1}H-^{1}H COSY (700 MHz, DMSO-d_6) spectrum of compound 4.

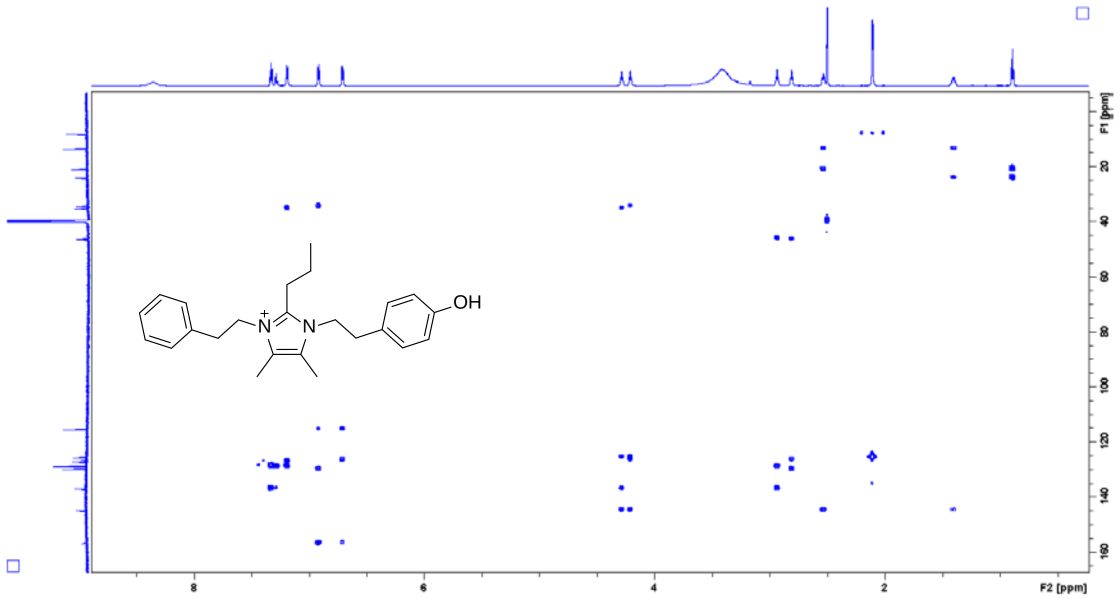

Figure S31. The ^{1}H-^{13}C HMBC (700 MHz, DMSO-d_6) spectrum of compound 4.

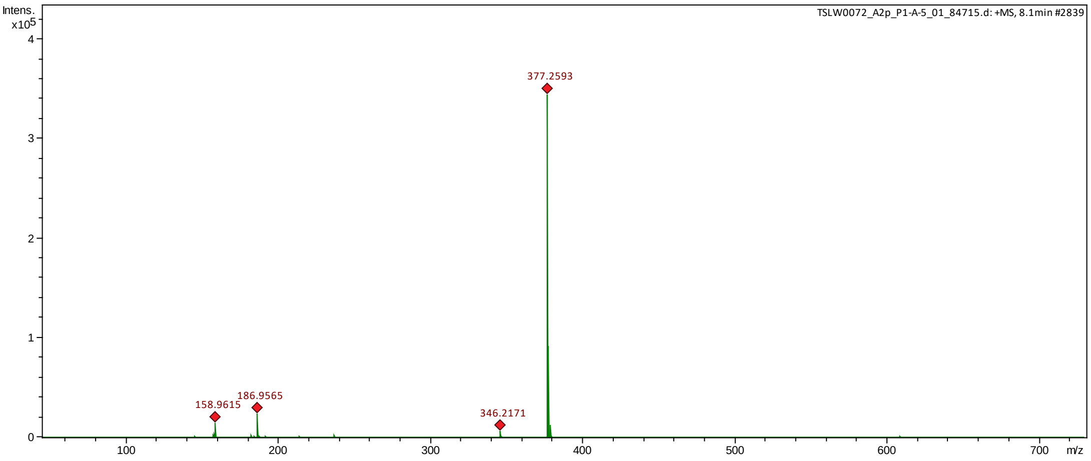

Meas. m/z	#	Ion Formula	m/z	err [ppm]	mSigma	# mSigma	Score	rdb	eÂ¯ Conf	N-Rule
377.2593	1	C25H33N2O	377.2587	-1.5	8.8	1	100.00	10.5	even	ok

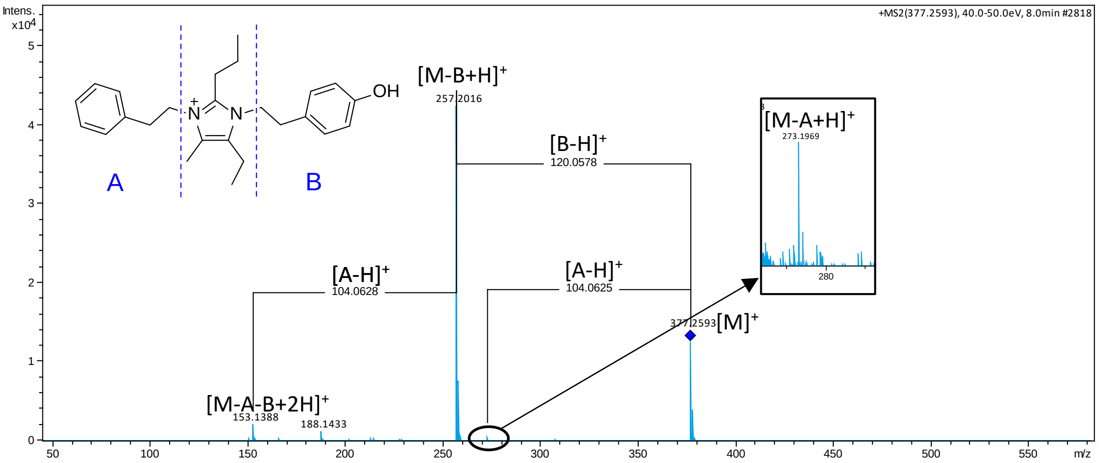

Figure S32. The HR-ESI-MS and HR-ESI-MS/MS of compound 5.

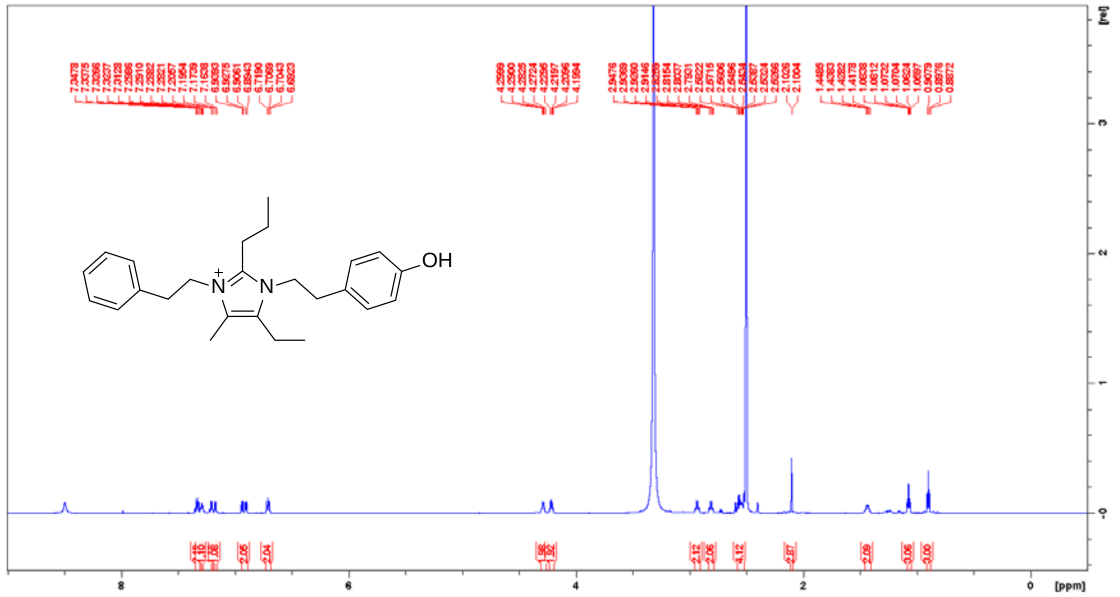

Figure S33. The ^{1}H-NMR (700 MHz, DMSO-d_6) spectrum of compound 5

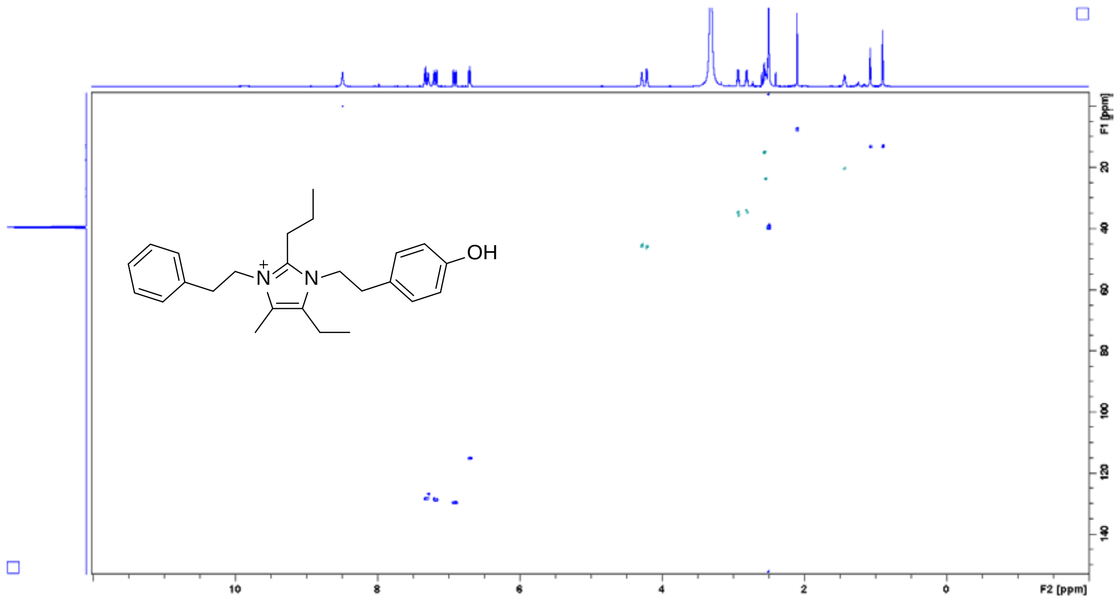

Figure S34. The HSQC (700 MHz, DMSO-d_6) spectrum of compound 5.

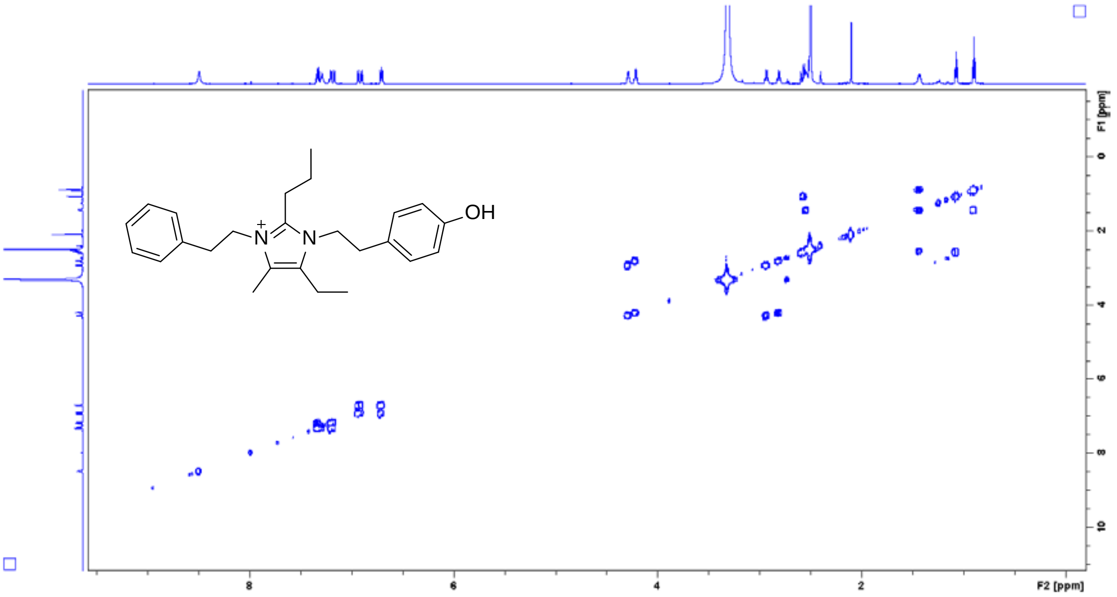

Figure S35. The ^{1}H-^{1}H COSY (700 MHz, DMSO-d_6) spectrum of compound 5.

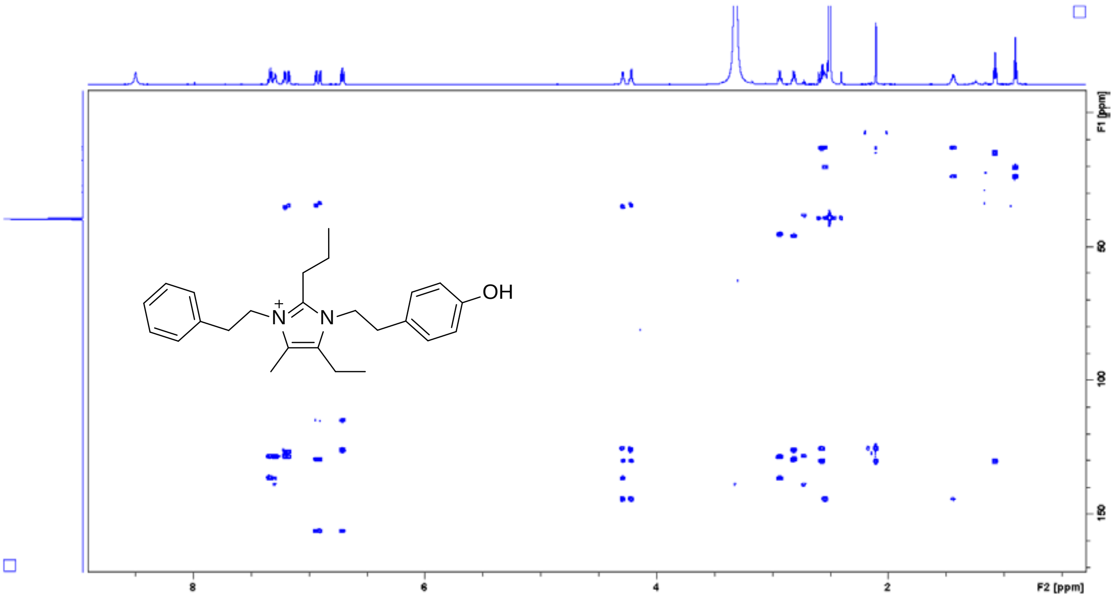

Figure S36. The ^{1}H-^{13}C HMBC (700 MHz, DMSO-d_6) spectrum of compound 5.

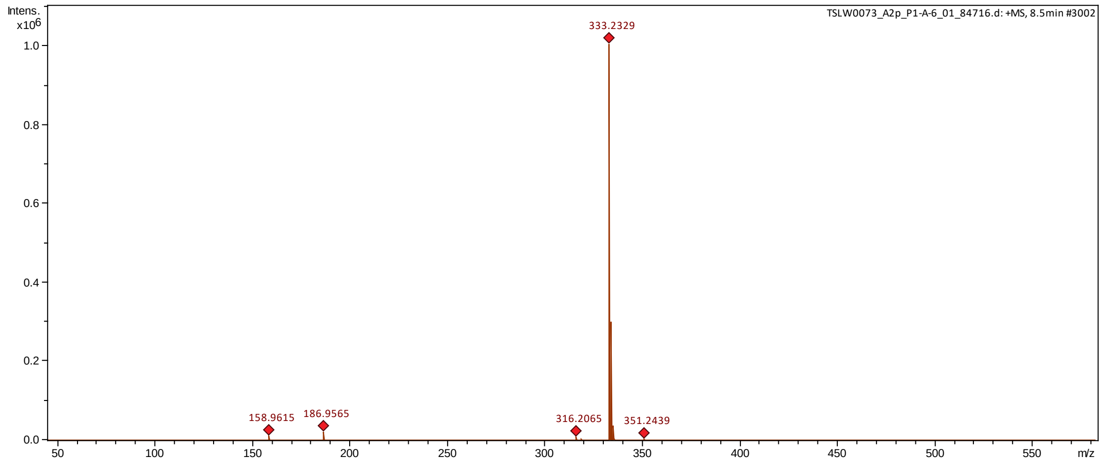

Meas. m/z	#	Ion Formula	m/z	err [ppm]	mSigma	# mSigma	Score	rdb	eÂ¯ Conf	N-Rule
333.2329	1	C23H29N2	333.2325	-1.2	23.6	1	100.00	10.5	even	ok

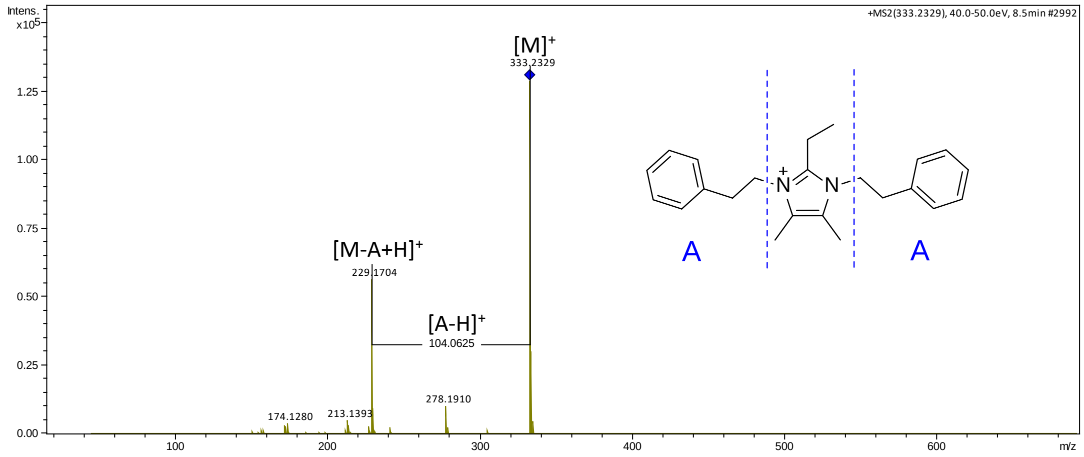

Figure S37. The HR-ESI-MS and HR-ESI-MS/MS of compound 6.

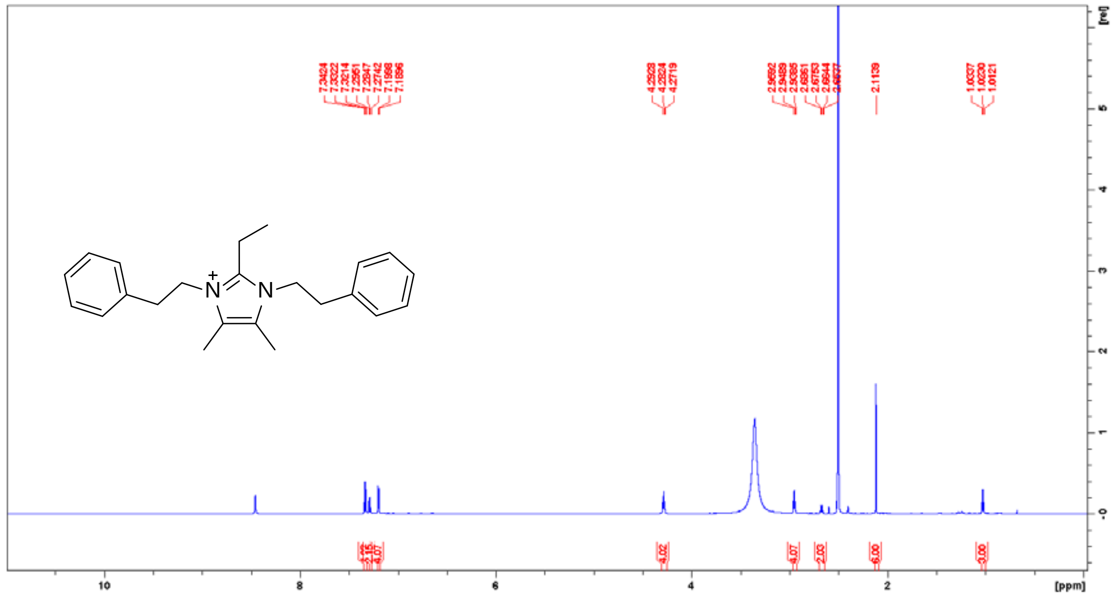

Figure S38. The ^{1}H-NMR (700 MHz, DMSO-d_6) spectrum of compound 6

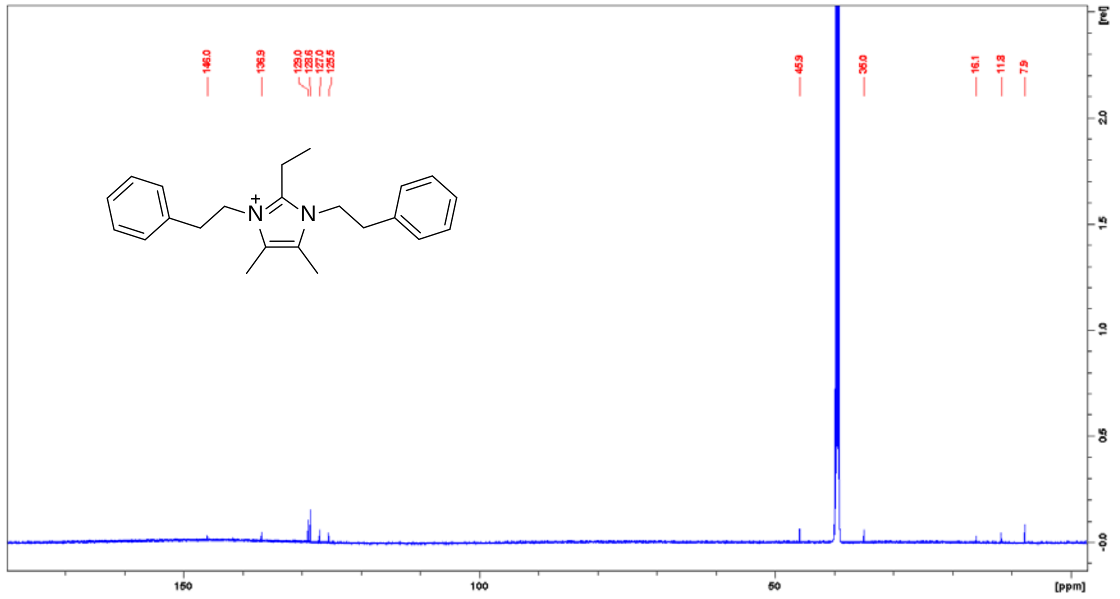

Figure S39. The ^{13}C-NMR (175 MHz, DMSO-d_6) spectrum of compound 6

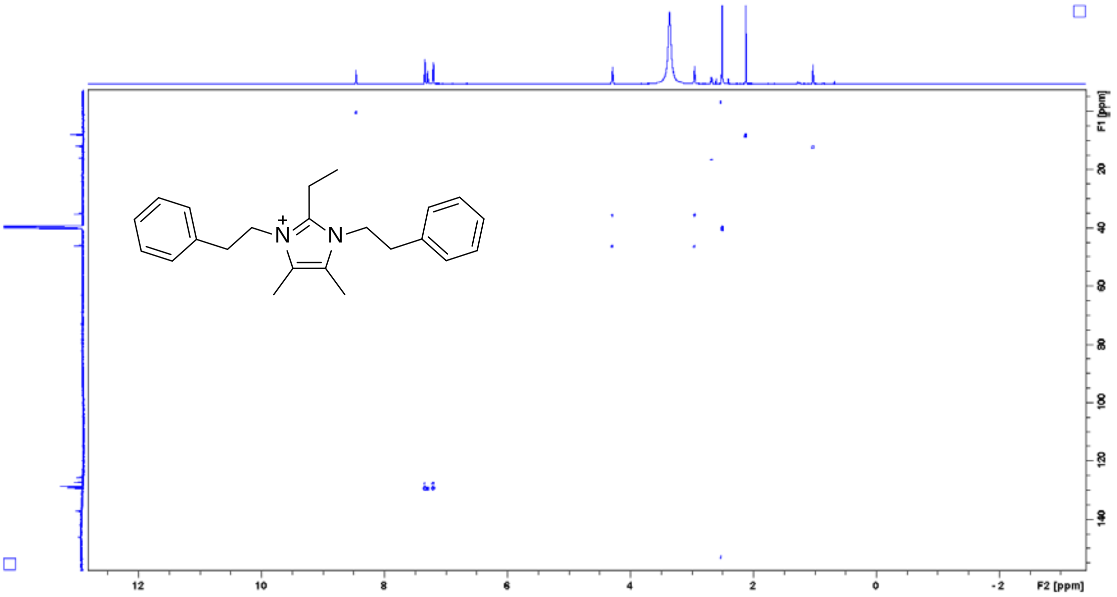

Figure S40. The HSQC (700 MHz, DMSO-d_6) spectrum of compound 6.

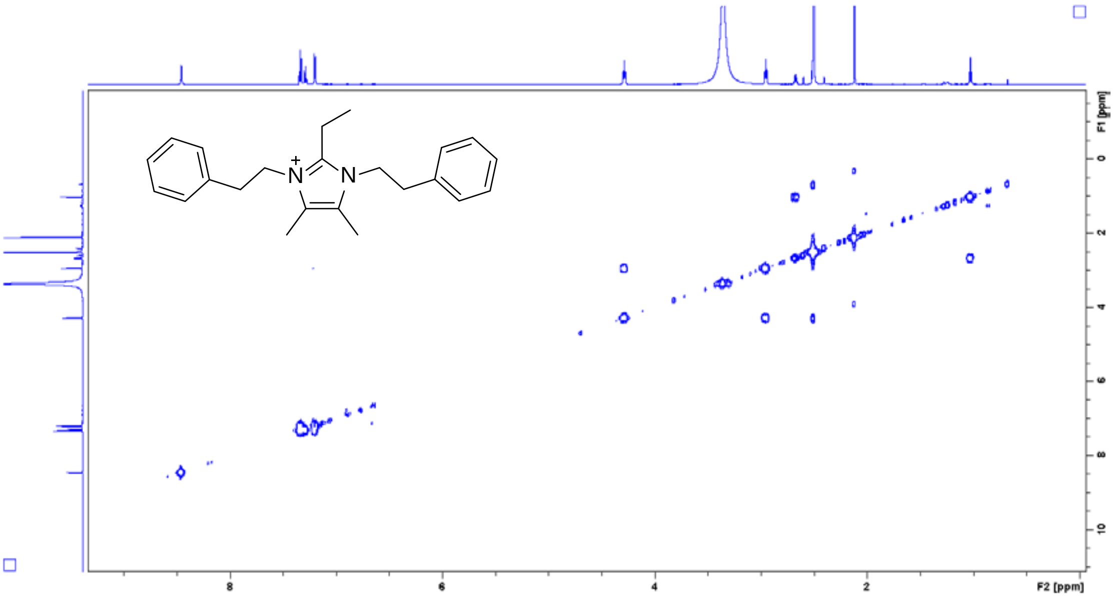

Figure S41. The ^{1}H-^{1}H COSY (700 MHz, DMSO-d_6) spectrum of compound 6.

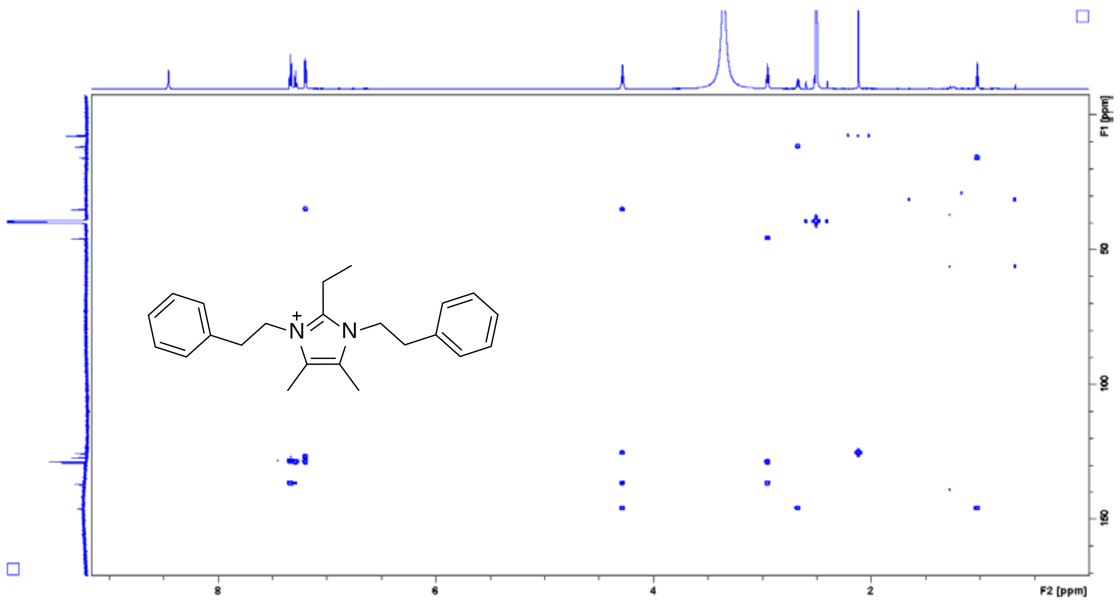

Figure S42. The ^{1}H-^{13}C HMBC (700 MHz, DMSO-d_6) spectrum of compound 6.

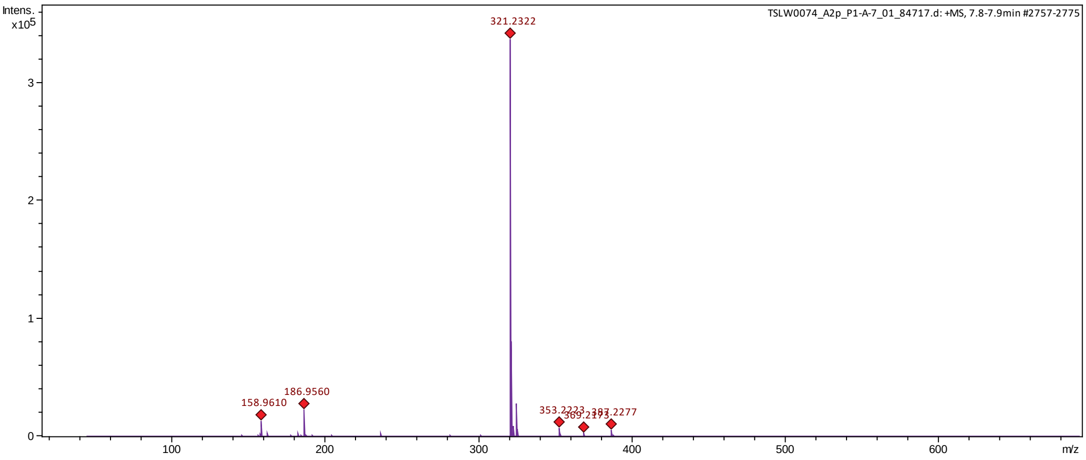

Meas. m/z	#	Ion Formula	m/z	err [ppm]	mSigma	# mSigma	Score	rdb	eÂ¯ Conf	N-Rule
321.2322	1	C22H29N2	321.2325	1.0	4.4	1	100.00	9.5	even	ok

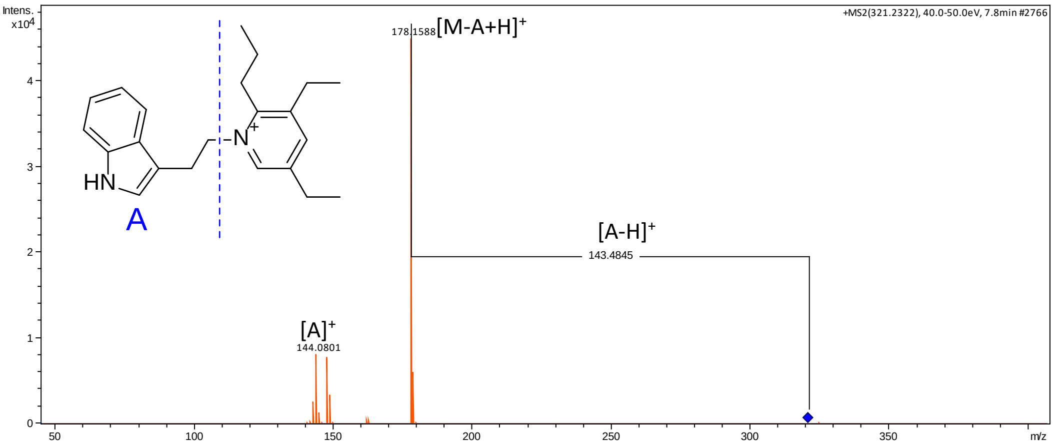

Figure S43. The HR-ESI-MS and HR-ESI-MS/MS of compound 7.

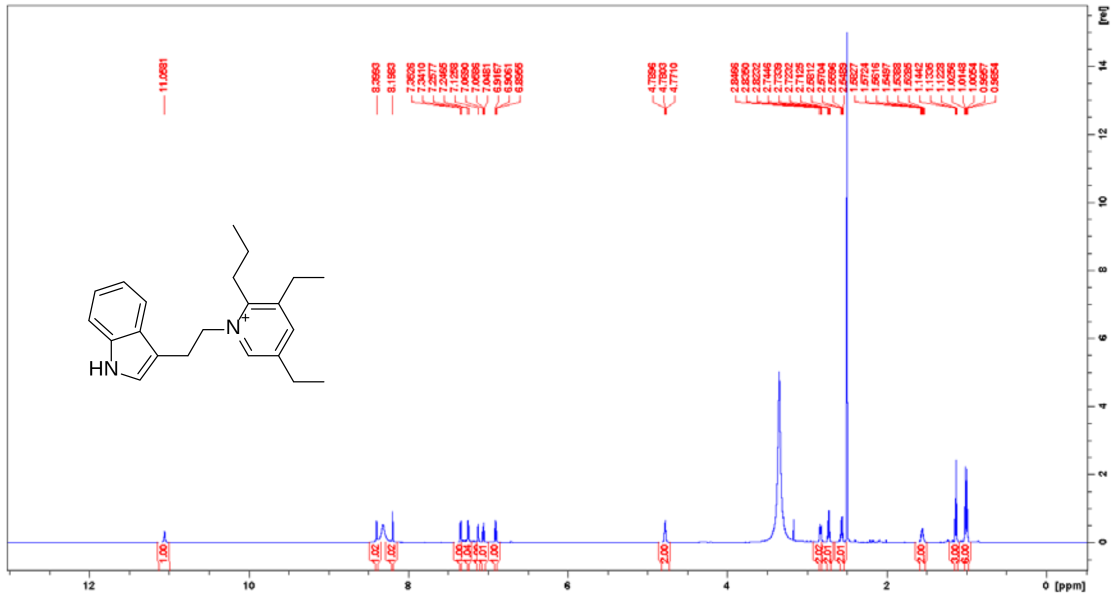

Figure S44. The ¹H-NMR (700 MHz, DMSO-d_6) spectrum of compound 7

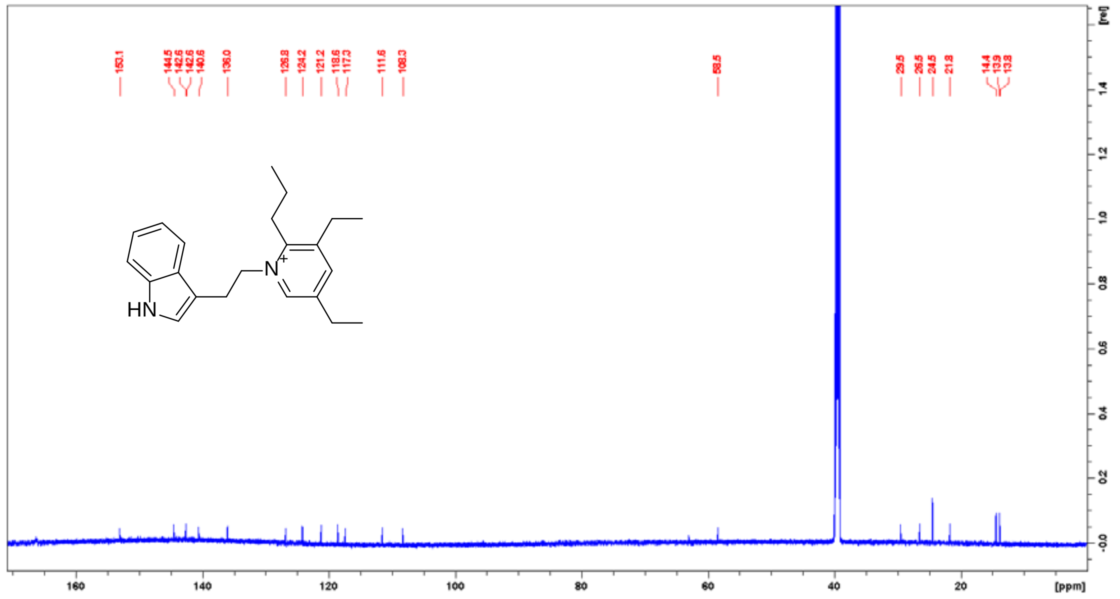

Figure S45. The ¹³C-NMR (175 MHz, DMSO-d_6) spectrum of compound 7

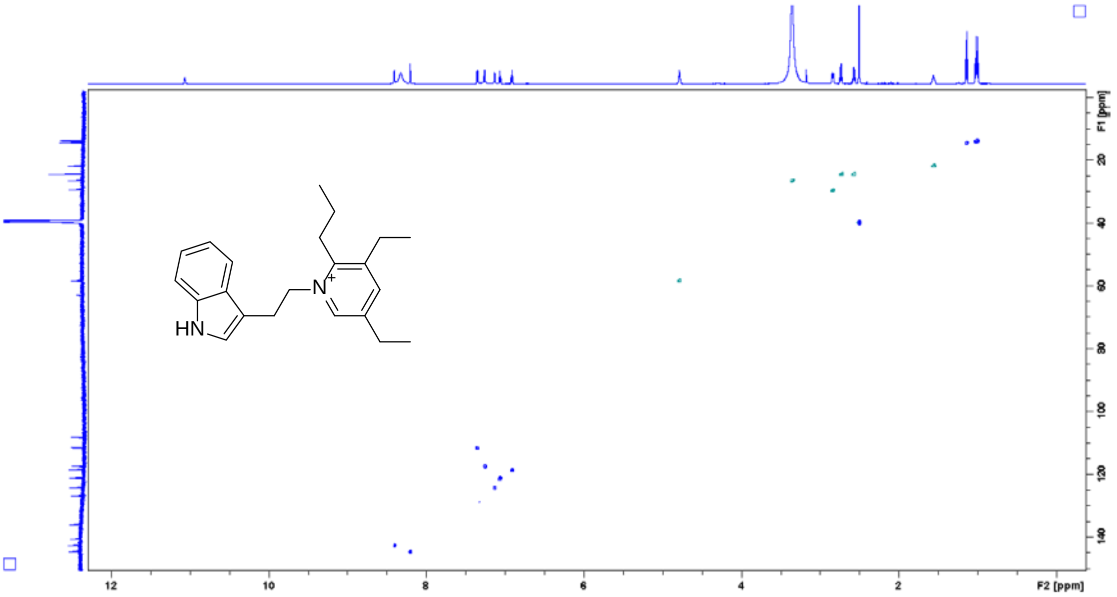

Figure S46. The HSQC (700 MHz, DMSO-d_6) spectrum of compound 7.

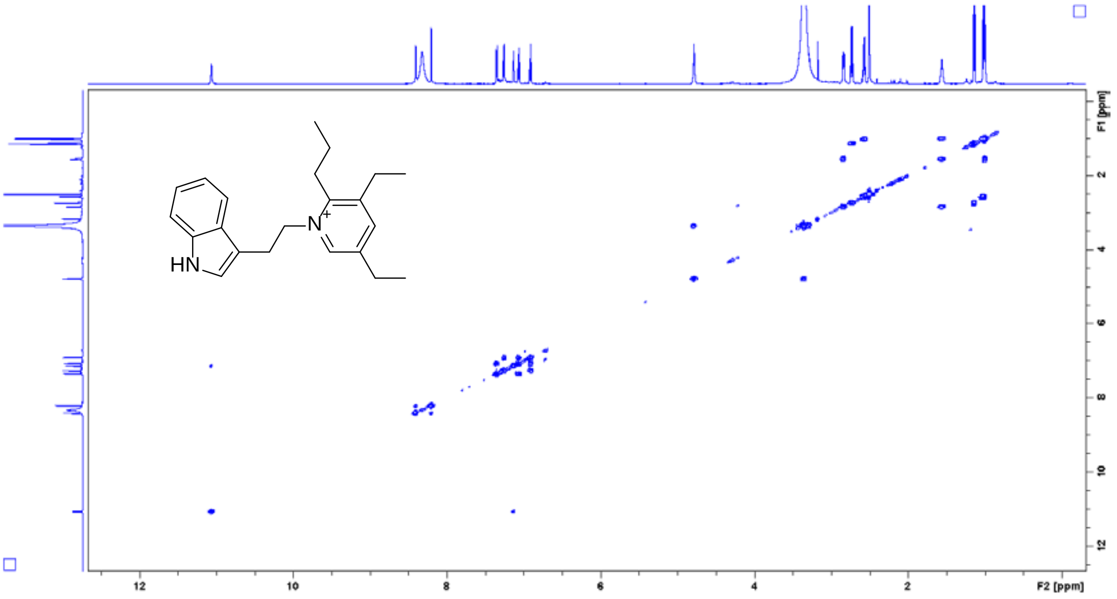

Figure S47. The ^{1}H-^{1}H COSY (700 MHz, DMSO-d_6) spectrum of compound 7.

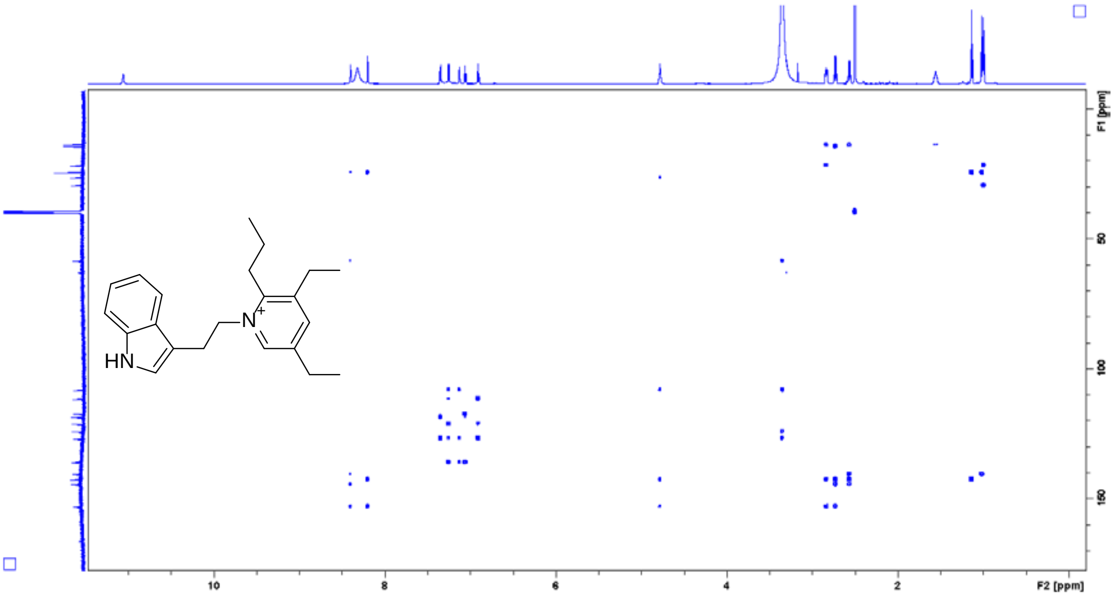

Figure S48. The ^{1}H-^{13}C HMBC (700 MHz, DMSO-d_6) spectrum of compound 7.

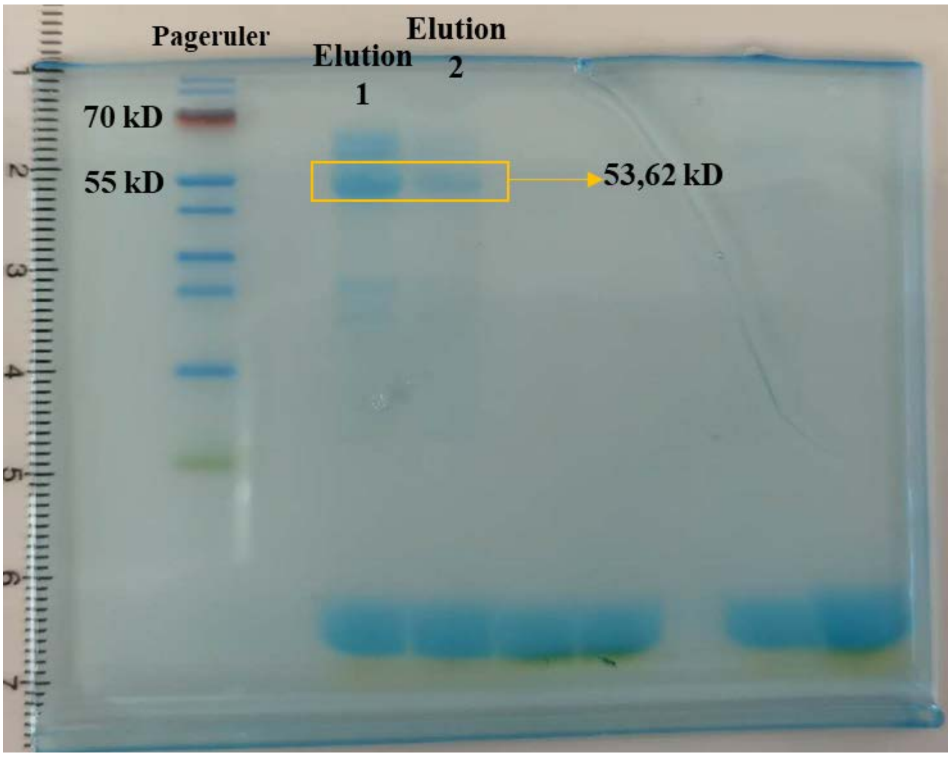

Figure S49. SDS-PAGE gel showing the purification of His-tagged DisA.
